# Mutational Slime Mould Algorithm for Gene Selection

**DOI:** 10.3390/biomedicines10082052

**Published:** 2022-08-22

**Authors:** Feng Qiu, Pan Zheng, Ali Asghar Heidari, Guoxi Liang, Huiling Chen, Faten Khalid Karim, Hela Elmannai, Haiping Lin

**Affiliations:** 1 Department of Computer Science and Artificial Intelligence, Wenzhou University, Wenzhou 325035, China; 2 Information Systems, University of Canterbury, Christchurch 8014, New Zealand; 3 Department of Information Technology, Wenzhou Polytechnic, Wenzhou 325035, China; 4 Department of Computer Sciences, College of Computer and Information Sciences, Princess Nourah bint Abdulrahman University, P.O. Box 84428, Riyadh 11671, Saudi Arabia; 5 Department of Information Technology, College of Computer and Information Sciences, Princess Nourah bint Abdulrahman University, P.O. Box 84428, Riyadh 11671, Saudi Arabia; 6 Department of Information Engineering, Hangzhou Vocational & Technical College, Hangzhou 310018, China

**Keywords:** gene selection, slime mould algorithm, Cauchy mutation, crossover and mutation, medical diagnosis

## Abstract

A large volume of high-dimensional genetic data has been produced in modern medicine and biology fields. Data-driven decision-making is particularly crucial to clinical practice and relevant procedures. However, high-dimensional data in these fields increase the processing complexity and scale. Identifying representative genes and reducing the data’s dimensions is often challenging. The purpose of gene selection is to eliminate irrelevant or redundant features to reduce the computational cost and improve classification accuracy. The wrapper gene selection model is based on a feature set, which can reduce the number of features and improve classification accuracy. This paper proposes a wrapper gene selection method based on the slime mould algorithm (SMA) to solve this problem. SMA is a new algorithm with a lot of application space in the feature selection field. This paper improves the original SMA by combining the Cauchy mutation mechanism with the crossover mutation strategy based on differential evolution (DE). Then, the transfer function converts the continuous optimizer into a binary version to solve the gene selection problem. Firstly, the continuous version of the method, ISMA, is tested on 33 classical continuous optimization problems. Then, the effect of the discrete version, or BISMA, was thoroughly studied by comparing it with other gene selection methods on 14 gene expression datasets. Experimental results show that the continuous version of the algorithm achieves an optimal balance between local exploitation and global search capabilities, and the discrete version of the algorithm has the highest accuracy when selecting the least number of genes.

## 1. Introduction

Microarray technology [1,2] is a new analytical tool that simultaneously measures the expression levels of thousands of genes in a single experiment, greatly helping researchers understand disease at the genetic level. However, the gene expression data are all high-dimensional, and the number of features is much larger than the number of samples [3,4]. A large number of unrelated and complex features will reduce the computational performance and waste computational resources, which is not conducive to the classification of gene expression [5,6,7]. The application of feature selection in genes, namely gene selection, is a screening technology to reduce unrelated genes and gene dimensions [8,9,10]. Through this technology, feature size can be effectively reduced, and classification performance can be improved [11,12,13].

Feature selection is an essential technology in data processing and machine learning [7,14]. The essence is to pick out the relatively optimal features from the raw data so that the data go from high to low dimensions [15,16]. The commonly used (classical) feature selection methods can be divided into filter, wrapper, embedded, and hybrid methods [17]. Filter methods typically select features independently and evaluate individual features without providing a practical evaluation across feature subsets, which may ignore the correlation between feature combinations [18,19,20,21]. Because it does not use any algorithm, the computation is less, leading to the failure to find the optimal gene subset. The wrapper method relies on the classification algorithm to select the feature subset, which can obtain the ideal effect, but the calculation cost is high [22,23,24]. Embedded methods usually use some machine learning algorithms and models for training and then select the best feature subset through the classifier algorithm [25]. When extracting features, it needs to train the model to automatically obtain the corresponding threshold value, which is realized by the algorithm with a built-in feature selection method. The hybrid method combines the advantages of the filter and wrapper methods to determine the optimal subset of a given cardinality by independent measurement and select the final subset in the optimal subset using a mining algorithm [26,27,28,29].

Optimization methods can be approximated or deterministic [30], and their model can be single objective and multi-objective, including multiple objective algorithms that can deal with multiple objectives at once [31,32]. In recent years, since the wrapper method based on the meta-heuristic algorithm or its variants can find an acceptable solution, that is, the approximate optimal subset of features, it has been widely used in feature selection [33,34]. In this study, we tried to use an improved slime mould algorithm (SMA), called ISMA, to develop an efficient wrapper gene selection method for finding the smallest feature subset. The optimization algorithm proposed in this paper is aimed at the shortcomings and characteristics of the original SMA, using the main operators of the SMA, but some of the operators use binary conversion to adapt to the genetic selection problem because the original version of the algorithm was created to solve the continuity problem. SMA is a new meta-heuristic algorithm recently proposed by Li et al. [35], which is used to deal with continuous global optimization and engineering design problems. It is an optimal algorithm used to simulate the dynamic vibration behavior of slime mould in dispersive foraging and food searching. This method consists of three search patterns with different morphologic variations, which are mathematically expressed using a unique model. The mathematical model of SMA mainly adopts the adaptive weight to simulate the propagation wave of the biological oscillator and generates positive feedback during the optimization process, which helps form an optimal exploration trajectory of the optimal solution with good searchability. In addition, the survey and results confirm that SMA achieves a balanced competitive advantage between global exploration and local exploitation. Notably, it shows a superior tendency towards local exploitation. With the help of adaptive weighting and efficient and reasonable structure, SMA can provide significantly enhanced performance compared to many recognized advanced algorithms, such as whale optimization algorithm (WOA), gray wolf optimization (GWO), grasshopper optimization algorithm (GOA), moth-flame optimization (MFO), ant lion optimizer (ALO), bat algorithm (BA), salp swarm algorithm (SSA), sine cosine algorithm (SCA), particle swarm optimization (PSO), and differential evolution (DE) [36]. Other examples include biogeography-based learning particle swarm optimization (BLPSO) [37], comprehensive learning particle swarm optimizer (CLPSO) [38], improved grey wolf optimization (IGWO)[39], and binary whale optimization algorithm (BWOA) [40], etc. Therefore, SMA has been applied in engineering design problems [35,41], solar photovoltaic cell parameter estimation [42,43], multi-spectral image segmentation [44], numerical optimization [45], prediction problems [46,47], support vector regression parameter adjustment [48] and other aspects. This algorithm is a sufficiently effective meta-heuristic optimization algorithm, but it may have the shortcoming of local optimal convergence and slow convergence speed when dealing with some complex problems. Therefore, there are some challenges in improving the optimization capability of SMA and expanding its application value.

In order to alleviate the shortcomings of traditional SMA and strengthen the trend of coordination between global exploration and local exploitation, an advanced SMA variant was proposed based on the reasonable integration of Cauchy mutation (CM) and crossover mutation (MC). After the initial search agent is generated, the solution is updated in three phases. First, execute the search process of SMA and update the search agent. The Cauchy mutation strategy is adopted in the second stage to adjust the SMA-based search agent. Finally, the optimal search agent is selected from the previous generation of search agents through a crossover mutation strategy. In addition, we convert the continuous version of ISMA to a discrete ISMA with a transfer function. Tests on gene expression data sets have shown that BISMA has significant advantages over some advanced gene selection methods and is very effective. It shows that ISMA can effectively solve high-dimensional complex gene problems, which makes improving SMA more valuable.

The main contributions in this paper can be summarized as follows:

(1) An improved slime mould algorithm (ISMA) is proposed to solve continuous global optimization problems and high-dimensional gene selection problems.
(2) The performance of the ISMA algorithm is verified by comparing it with several famous optimization algorithms.
(3) Different transfer functions are used to transform the proposed ISMA into a discrete version of BISMA, and they are compared to choose the most suitable transfer function for the binary ISMA optimizer.
(4) The optimal BISMA version was selected as a gene selection optimizer to select the optimal gene subset from the gene expression data set.
(5) The performance of the selected method is verified by comparing it with several other advanced optimizers.

The rest of this article is as follows: The second part introduces the work of gene selection and meta-heuristic algorithms. In the third section, Cauchy mutation and a crossover mutation strategy based on the DE algorithm are introduced in detail, and ISMA is proposed. In the fourth section, a series of comparative experiments between ISMA and other similar algorithms are introduced. In the fifth part, we design the structure of wrapper gene selection for discrete ISMA. In the sixth part, we discuss the application of BISMA and other related algorithms in gene selection. In the seventh part, we discuss a summary of the proposed work as well as its shortcomings and implications. The eighth part gives a brief description of the work of this paper and points out the future direction of the work.

## 2. Related Works

The dimensions of microarray data are often extremely asymmetric and highly redundant, and most genes are considered to be irrelevant to the category under study. Traditional classification methods cannot effectively process such data. Many researchers have achieved good results using machine learning techniques to process gene expression data sets.

### 2.1. Machine Learning for Gene Selection

Singh et al. [49] proposed a hybrid improved chaotic emperor penguin (CEPO) algorithm based on the Fisher criterion, ReliefF, and extreme learning machine (ELM) for microarray data analysis. In this paper, the Fisher criterion and ReliefF method were first used as gene selection filters, and then relevant data were used to train the ELM to obtain a better model. Banu et al. [50] used the fuzzy clustering method to assign initial values to each gene and then predicted the likelihood of belonging to each cluster to carry out gene selection. The comparative experimental results show that the fuzzy clustering algorithm performs well in gene prediction and selection. Chen et al. [51] proposed a support vector machine for binary tumor diagnosis, extending the three kinds of support vector machines to improve the performance of gene selection. At the same time, lasso, elastic net, and other sparse regression methods were introduced for cancer classification and gene selection. Mahendran et al. [52] conducted an extensive review of recent work on machine learning-based selection and its performance analysis, classified various feature selection algorithms under supervised, unsupervised and semi-supervised learning, and discussed the problems in dealing with high and low sample data. Tan et al. [53] proposed an integrated machine learning approach to analyze multiple gene expression profiles of cervical cancer to find the genomes associated with it, with the expectation that it could help in diagnosis and prognosis. The gene expression data were identified effectively through the analysis of three steps.

Zhou et al. [54] proposed an improved discretized particle swarm optimization algorithm for feature selection. In their work, a modest pre-screening process is first applied to obtain fewer features; then, a better cutting combination is found through the encoding and decoding method based on PSO and the local search strategy guided by probability to obtain the desired feature subset. Zohre Sadeghian et al. [55] proposed a three-stage feature selection method based on the S-BBOA algorithm. In the first stage, the minimum redundancy—maximum new classification information (MRMNCI) feature selection was used to remove 80% of the irrelevant and redundant features. The best feature subset was chosen using IG-BBOA in the second step. Furthermore, the similarity ranking approach was used to choose the final feature subset. Veredas Coleto-Alcudia et al. [56] proposed a new hybridization method based on the dominance degree artificial bee colony algorithm (ABCD) to investigate the problem of gene selection. The method combines the first step of gene screening with the second part of the optimization algorithm to find the optimal subset of genes for the classification task. The first step is to use the Analytic Hierarchy Process (AHP) to select the most relevant genes in the dataset through five sequencing methods. In this way, gene filtering reduces the number of genes that need to be managed. For the second step, gene selection can be divided into two objectives: minimizing the number of selected genes and maximizing classification accuracy. Lee et al. [57] embedded the formal definition of correlation into Markov coverage (MB) and established a new multi-feature sequencing method, which was applied to high-dimensional microarray data, enhancing the efficiency of gene selection and, as a result, the accuracy of microarray data classification.

### 2.2. Swarm Intelligence for Gene Selection

Alok Kumar Shukla et al. [4] created TLBOGSA, a hybrid wrapper approach that combines the features of the Teaching Learning based Optimization (TLBO) and the Gravity Search Algorithm (GSA). TLBOGSA was updated with a new encoding approach that transformed the continuous search space into the binary search space, resulting in the binary TBSA. First, significant genes from the gene expression dataset were chosen using the minimal redundancy and maximum correlation (mRMR) feature selection approach. Then, using a wrapper strategy, information genes were chosen from the reduced data generated by the mRMR. They developed the gravitational seeking mechanism in the teaching stage to boost the evolutionary process’s searching capabilities. The technique selected the most reasonable genes using a Naive Bayes classifier as a fitness function, which is useful for accurate cancer classification. Based on the phase diagram approach, Elahe Khani et al. [58] suggested a unique gene selection algorithm, and Ridge logistic regression analysis was performed to evaluate the likelihood that the genes belong to a stable group of genes with excellent classification ability. To address the problems, a methodology for the final selection of the selected set is suggested. The model’s performance was assessed using the B632+ error estimation approach. To identify genes from gene expression data and valuable information genes from cancer data genes, a decision tree optimizer based on particle swarm optimization was presented by Chen et al. [59]. Experimental results demonstrate that this strategy outperforms different popular classifiers, including support vector machines, self-organizing mapping, and back propagation neural networks. Dabba et al. [10] developed the Quantum MFO (QMFOA), a swarm intelligent gene selection technique based on the fusion of quantum computing with the MFO, to discover a relatively small subset of genes for high-precision sample classification. The QMFOA gene selection algorithm has two stages: the first is preprocessing, which acquires a preprocessing gene set by measuring the redundancy and correlation of genes, and the second is hybrid combination and gene selection, which utilizes several techniques such as MFO, quantum computing, and support vector machine. To select a limited, representative fraction of cancer-related genetic information, Mohamad et al. [60] developed an enhanced binary particle swarm optimization for gene selection. The velocity of particles is incorporated in this approach to give the rate of particle position change, and the particle position update rule is presented. The experimental findings show that the suggested technique outperforms the classic binary PSO in terms of classification accuracy while picking fewer genes (BPSO).

## 3. The Proposed ISMA

### 3.1. SMA

Several swarm intelligence optimization techniques have appeared successively in recent years, such as slime mould algorithm (SMA) [35], Harris hawks optimization (HHO) [61], hunger games search (HGS) [62], Runge Kutta optimizer (RUN) [63], colony predation algorithm (CPA) [64], and weighted mean of vectors (INFO) [65]. Due to the simplicity and efficiency of swarm intelligence algorithms, they have been widely used in many different fields, such as image segmentation [66,67], the traveling salesman problem [68], feature selection [69,70], practical engineering problems [71,72], fault diagnosis [73], scheduling problems [74,75,76], multi-objective problems [77,78], medical diagnosis [79,80], economic emission dispatch problems [81], robust optimization [82,83], solar cell parameter identification [84], and optimization of machine learning models [85]. Among them, SMA is a new bionic stochastic optimization problem, simulating slime mold behavior and morphological changes during foraging. At the same time, SMA used weight to simulate the positive and negative feedback effects of slime mould propagation waves during foraging behavior to construct a venous network with different thicknesses. The morphology of the slime mould changed with the three search patterns: proximity to food, wrap around food, and oscillation.

**Figure 1:**
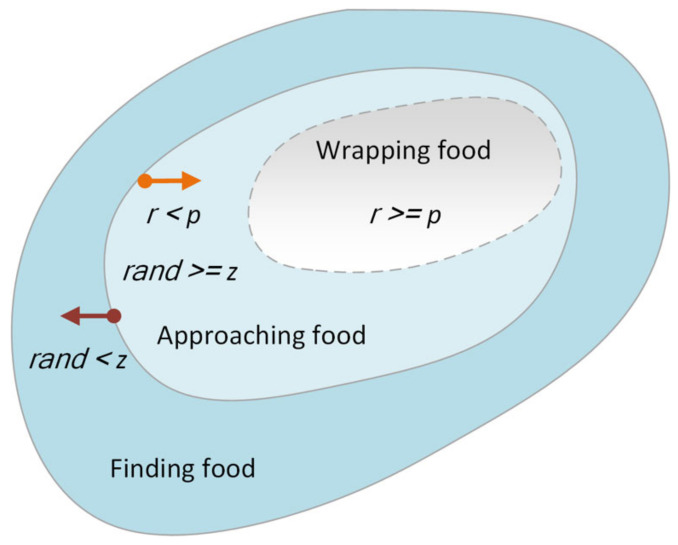
A brief description of SMA.

From the brief description of SMA shown in Figure 1, the random value rand helps to find the optimal solution. The slime moulds were randomly distributed in any direction to search for solutions (food), and when rand<z, there was no venous structure. During the search phase, when rand≥z and r<p, individuals form diffuse venous structures to access food. The adaptive change of decision parameter p ensures better adaptability of the transition from the exploration stage to the exploitation stage. During the exploitation phase, when r≥p, the individual encapsulates the solution (food) through venous fibrillation.

Based on the following significant parameters, a specific mathematical model of SMA can be constructed to represent the three contraction modes of slime mould:

(1) $$X\left(t+1\right)=\left\{\begin{array}{cc} rand\cdot \left(UB-LB\right)+LB, & rand<z\\ X_{b}\left(t\right)+vb\left(t\right)\cdot \left(W\cdot X_{A}\left(t\right)-X_{B}\left(t\right)\right), & r<p\\ vc\left(t\right)\cdot X\left(t\right), & r\geq p \end{array}\right.$$

where $X\left(t\right)$ and $X\left(t+1\right)$ represent the position vectors of slime mould during iteration t and $\left(t+1\right)$, respectively. UB and LB indicate the upper and lower boundaries of the search space, respectively. $X_{b}$ denotes the position vector of the individual with the highest fitness (highest concentration). $X_{A}\left(t\right)$ and $X_{B}\left(t\right)$ indicate the position vectors of random individuals selected from the slime mould during iteration t. rand and r are random values between 0 and 1. The parameter z is set to 0.03 as in the original literature.

In addition, the decision parameter p can be calculated as follows:

(2) $$p=tanh\;tanh\;\left|S\left(i\right)-DF\right|$$

where $S\left(i\right)$ indicates the fitness of the ith individual in the slime mould X, i∈1,2, ⋯,N. N. denotes the size of the population. DF represents the best fitness, which is attained during all of the iterations.

W is the weight vector of slime mould, which can be obtained from the following equation. This vector mimics the rate at which slime mould shrinks around food for different food masses.

(3) $$W\left(SmellIndex\left(i\right)\right)=\left\{\begin{array}{c} 1+r\cdot \log\left(\frac{bF-SmellOrder\left(i\right)}{bF-wF}+1\right),\;condition\\ 1-r\cdot \log\left(\frac{bF-SmellOrder\left(i\right)}{bF-wF}+1\right),\;otherwise \end{array}\right.$$

$$\left[SmellOrder,SmellIndex\right]=sort\left(S\right)$$

where bF and wF are the best and worst fitness obtained in the current iteration, respectively. SmellIndex and SmellOrder denote, respectively, the fitness sort order (smallest problems sorted in ascending order) and the corresponding fitness value. condition indicates the first half of SmellOrder and is also the overall fitness ordering value. condition simulates the individuals adjusting their search patterns dynamically according to the quality of things.

The collaborative interaction between the parameters vb and vc can simulate the selection behavior of slime mould. vb denotes a random value in the interval $\left[-a,a\right]$. The parameter vc represents a decrease in the number of iterations within the interval $\left[-b,b\right]$.

(4) $$a=\mathrm{arctanh}\left(1-\left(\frac{t}{Max\_iter}\right)\right)$$

(5) $$b=1-\left(\frac{t}{Max\_iter}\right)$$

where Max_iter indicates the maximum number of iterations.

The simplified pseudo-code of SMA is listed in Algorithm 1. We can find more specific descriptions in the original literature.
**Algorithm 1:** Pseudo-code of SMABegin Initialize the parameters: Max_iter, N
 Initialize slime mould population X
 While *t *≤ Max_iter  Calculate the fitness of each individual in the slime mould   Update best fitness and the $X_{b}$  Calculate the weight W according to Equation (3)  Calculate a according to Equation (4)  Calculate b according to Equation (5)  For i=1,2, ⋯,N (each search agent)   Update p according to Equation (2)   Update vb, vc based on a and b, respectively   Update the positions according to Equation (1)  EndFor   iteration = iteration + 1  EndWhile  Return the best fitness and $X_{b}$ End

### 3.2. The Cauchy Mutation Operator

In this section, we will briefly introduce the Cauchy mutation. The Cauchy density function can be described as:

(6) $$f_{t}\left(x\right)=\frac{1}{\pi }\frac{t}{t^{2}+x^{2}}\;,-\infty <x<\infty$$

where t>0 and is the proportional parameter, and the distribution function is expressed as follows:

(7) $$F_{t}\left(x\right)=\frac{1}{2}+\frac{1}{\pi }arctan\left(\frac{x}{t}\right)$$

By increasing the search range in each generation, individuals can be guaranteed to find better solutions in a wider range, thus avoiding local optimization. Therefore, Cauchy mutation was selected as an improved mechanism.

In the original SMA based on Equations (6) and (7), the version using the Cauchy mutation operation is expressed as:

(8) $$x_{i\_cauchy}=x_{i}\times \left(1+Cauchy\right)$$

where Cauchy is the random number of the distribution obtained by the Cauchy distribution,$\;x_{i}$ is a position in the SMA at the time of the current iteration,$\;x_{i\_cauchy}$ is the corresponding position of $x_{i}$ after Cauchy mutation. The introduction of the Cauchy mutation mechanism improves the foraging behavior of slime mould searching the unknown space, so the quality of SMA solutions can be enhanced by using the Cauchy operator in the simulation process.

### 3.3. The Mutation and Crossover Strategy in DE

During the optimization procedure, the major operations are mutation and crossover. Each solution $x_{i}$ = {$x_{i1},x_{i2},x_{i3},\;\ldots \;,x_{in}\}$ is a vector of *n* dimensions.

A. Mutation

A mutant vector can be generated via the mutation operator ${?}_{i}$ according to selected components from randomly nominated vectors $x_{a}$, $x_{b}$, and $x_{c}$, where a ≠ b ≠ *c ≠* i. The mathematical equation can be represented as follows:

(9) $$u_{i}=x_{a}+F*\left(x_{b}-x_{c}\right)$$

where *F* is a random number that is able to control the mutation’s perturbation size.

B. Crossover

The crossover operator may construct a trial vector $v_{i}$ by applying crossover to a mutant vector, where the trial vector is constructed by randomly selecting items from the mutant $u_{i}$ and the target vector $x_{i}$ depending on the probability ratio $P_{c}$. The math formula appears such as this:

(10) $$v_{ij}=\left\{\begin{array}{l} u_{ij}\;;\;rand\leq P_{c}\;or\;j=j_{0}\\ x_{ij};\;otherwise \end{array}\right.$$

The probability feature $P_{c}$ controls the diversity of the swarm and relieves the risk of local optima, and $j_{0}$ is an index between [1,2,3, …, $N_{p}$], which guarantees that $v_{i}$ obtains at least one component from $u_{i}$.

### 3.4. The Hybrid Structure of the Proposed ISMA

**Figure 2:**
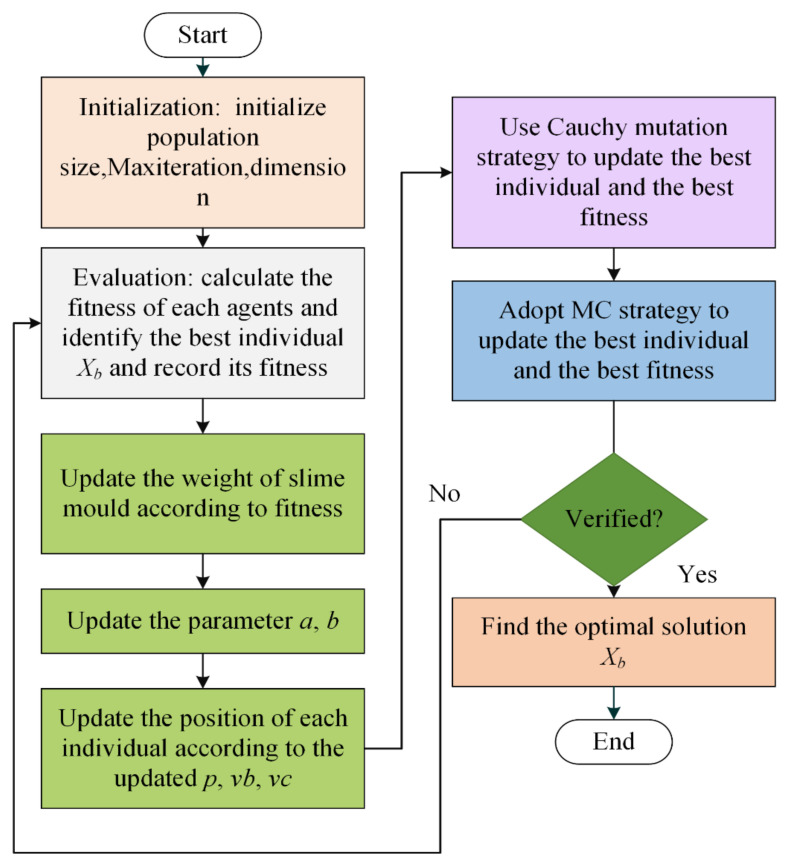
The framework of the proposed ISMA.

Considering that the original SMA may not converge to some suboptimal solutions precociously or face the risk of falling into local optimal solutions, the improved algorithm ISMA proposed in this paper combines two strategies, Cauchy mutation and crossover mutation based on DE, to promote the coordination of global exploration and local exploitation and forms a new SMA variant, namely ISMA. The structure of the proposed ISMA is shown in Figure 2, which is demonstrated in Algorithm 2. Under the ISMA framework, these two strategies are used, in turn, to generate the new search agent and the best agent with the best solution in the current iteration. Figure 2 depicts the ISMA process. As illustrated in the picture, the position of each agent may be rebuilt when the location of each agent is updated according to Equation (1), implying that each agent achieves the best solution in a larger search space.

The position update based on SMA is to solve the position vector of slime mould according to the optimization rules of SMA, as detailed in Section 2.1. This phase produces a population based on SMA. The Cauchy-based mechanism and the crossover mutation mechanism are based on the behavior of the Cauchy-based mutation operation and the crossover mutation operation shown in Section 2.2 to adjust the position vector of an SMA-based individual to produce a new SMA-based population. In this stage, the advantages of Cauchy and the crossover mutation mechanism in the exploration stage are utilized to make up for the shortcomings of the SMA exploration. Considering both mechanisms’ effects on search ability, this means increasing population size and thus population diversity. The research shows that this stage not only helps to promote the coordination of exploration and exploitation capabilities but also helps to improve the quality of solutions and accelerate the convergence rate.
**Algorithm 2:** Pseudo-code of ISMABegin Initialize of the parameters: Max_iter, N Initialize of slime mould population X While t≤Max_iter
  Calculate the fitness for each individual in slime mould   Update $X_{b}$ and the best fitness   Calculate the weight W,a,b according to Equations (3)–(5)   For i=1:N
   Update p using Equation (2)    Update vb, vc based on a and b, respectively    Update the positions by Equation (1)   EndFor   Use Cauchy mutation strategy to update the best individual and the best fitness   Adopt MC strategy to update the best individual and the best fitness  iteration = iteration + 1  EndWhile  Return the best fitness and $X_{b}$ as the best solution End

### 3.5. Computational Complexity

The proposed SMA structure mainly includes the following parts: initialization, fitness evaluation, fitness sorting, weight updating, position updating based on SMA strategy, position updating based on Cauchy mutation strategy, and position updating based on crossover mutation strategy, where N is the number of cells of slime mould, D is the dimension of function, and T is the maximum number of iterations. The computational complexity of initialization is $O\left(D\right)$. In the process of evaluation and sorting of fitness, the computational complexity is $O\left(N+NlogN\right)$. The computational complexity of updating the weight is $O\left(N\times D\right)$. The computational complexity of the location update process based on SMA is $O\left(N\times D\right)$. Similarly, the computational complexity of the location updating process based on the Cauchy mutation mechanism and cross mutation mechanism is $O\left(N\times D\right)$. Therefore, the total computational complexity of ISMA is $O(D+T\times N\times \left(1+4D+logN\right)$).

## 4. Experimental Design and Analysis of Global Optimization Problem

To evaluate successive versions of ISMA, we considered two experiments to compare the methods presented in this section with several other competitors. We used 23 continuous benchmark functions (including 7 unimodal functions, 6 multimodal functions, and 10 fixed-dimensional multimodal functions) and 10 typical CEC2014 benchmark functions (2 hybrid functions and 8 composition functions) for a total of 33 benchmark cases. Experiment 1 is a series of SMA variants with different update strategies: ISMA, CSMA, and MCSMA. The best SMA variants are obtained by comparing them with the original SMA and DE algorithm. Experiment 2 is to compare the ISMA algorithm with 8 other advanced optimization algorithms, including multi-population ensemble differential evolution (MPEDE) [86], successful history-based adaptive DE variants with linear population size reduction (LSHADE) [87], particle swarm optimization with an aging leader and challengers (ALCPSO) [88], comprehensive learning particle swarm optimizer (CLPSO) [38], chaos-enhanced sine cosine-inspired algorithm (CESCA) [89], improved grey wolf optimization (IGWO) [39], whale optimization algorithm with β-hill climbing (BHC) algorithm and associative learning and memory (BMWOA) [90], modified GWO with random spiral motions, simplified hierarchy, random leaders, oppositional based learning (OBL), levy flight (LF) with random decreasing stability index, and greedy selection (GS) mechanisms (OBLGWO) [91]. In this study, all experimental evaluations were conducted on a Windows 10(64-bit) operating system with 32GB RAM, Intel(R) Xeon(R) Silver 4110 CPU @ 2.40 GHz 2.10 GHz (dual processor), and MATLAB R2014a coding.

Table A1, Table A2, Table A3 and Table A4 contain information on 23 benchmark functions and 10 classic CEC2014 benchmark functions. It can be seen that the information of the 33 functions used in the experiment contains a wide variety of problems. These capabilities can be used not only to verify the local exploitation ability and global exploration ability but also to verify the ability to balance the two abilities. In addition, to reduce algorithm randomness’s impact on the experiment [92], we conducted 30 independent tests for each test case. In order to exclude the influence of other factors on the experiment, all the test algorithms were run under the same settings and conditions [93,94,95]. The maximum function evaluation was set as 300,000, and the population size was 30.

In addition, statistical results such as mean and standard deviation (std) are used to represent the global optimization ability and robustness of the evaluation method. The Wilcoxon signed-rank test at the significance level of 0.05 was used to measure the degree of improvement, which was statistically significant. It is worth noting that the label ‘+/=/−’ in the results indicates that ISMA is significantly superior to, equal to, or worse than other competitors. For a comprehensive statistical comparison, the Friedman test was used to see whether the performance of all the comparison algorithms on the baseline function differed and was statistically significant. The mean ranking value (ARV) of the Friedman test was used to evaluate the average performance of the investigated method. It is worth noting that a reliable comparison must involve more than 5 algorithms for more than 10 test cases [96].

### 4.1. Comparison between SMA Variant and Original SMA and DE Algorithm

**Figure 3:**
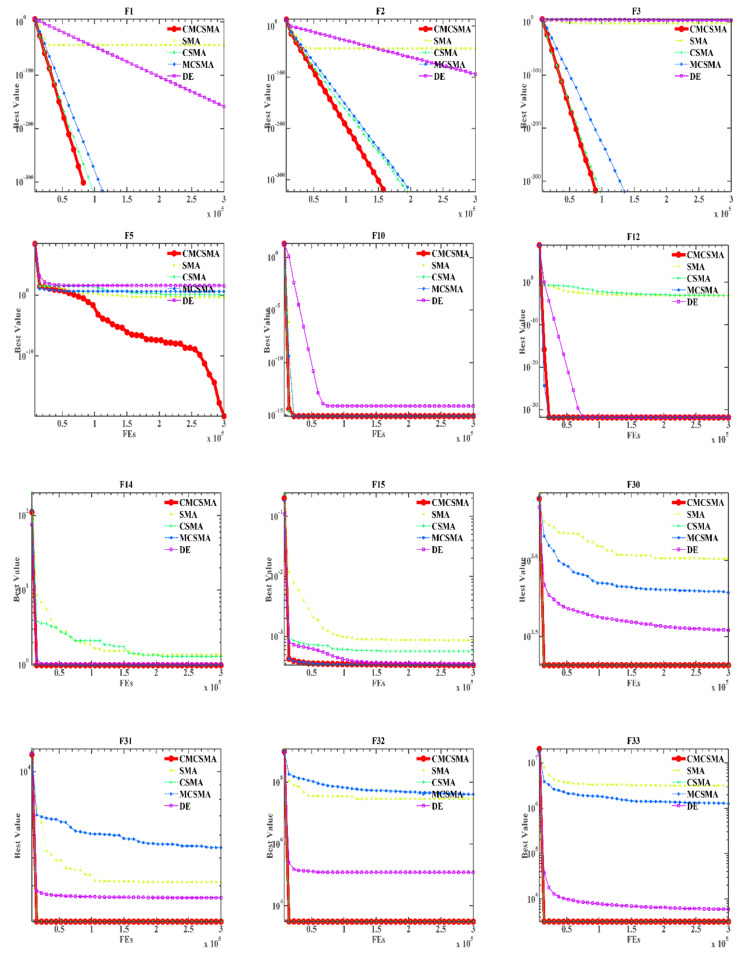
Convergence curves of the SMA variants and the original SMA and DE algorithms on twelve functions.

In this section, to prove the superiority of the Cauchy mutation mechanism and the combination of mutation and crossover strategies in DE, we compare the three combinations of the two mechanisms and the original SMA with the DE algorithm. The comparison results are shown in Table A5, Table A6 and Table A7, and the algorithm convergence curve is shown in Figure 3.

As the results show in Table A5 and Table A6, ISMA clearly outperforms the other mechanism combinations and the original SMA and DE algorithms, as ISMA outperforms almost all algorithms in handling most of the test functions. As can be seen from the ARV of Friedman’s test in Table A7, ISMA can be considered the first algorithm when comparing the five algorithms. Mean and std in Table A5 also indicate the superiority of ISMA in F1–F6, F9–F14, F26–28, and F30–33 functions. ISMA ranks 2nd in F7, F15–F17, F19–25, and F29. According to the statistical significance of *p*-values in Table A6, almost all values in the SMA column are less than 0.05, indicating that ISMA has significantly improved the original SMA algorithm. The final optimization effect of F1–3, F9–11 and F26–28 functions by CSMA and MCSMA is the same as that by ISMA. In summary, the results of the Wilcoxon signed-rank test show that, statistically, ISMA has significantly improved performance compared with other algorithms. The results show that the addition of the Cauchy mutation strategy and crossover mutation strategy based on DE is beneficial to ISMA’s exploitation ability and exploration ability and the balance between ISMA’s exploitation ability and exploration ability.

The convergence analysis can show which optimizer as an iterative method can reach better quality results within a shorter time [97,98]. Figure 3 shows the convergence curves of the comparison method on 12 functions. We can intuitively find that, compared with the original SMA, DE, and other two SMA variants, the ISMA using the two mechanisms has a better effect. Combining the two mechanisms makes the SMA avoid falling into the local optimal solution and can obtain the global optimal solution. The overall advantage of ISMA is significant because of the positive effect of the Cauchy mutation mechanism and the crossover mutation strategy on SMA, which highlights the optimization capability of the proposed method.

### 4.2. Comparison with Advanced Algorithms

In this experiment, we compare ISMA with several typical advanced algorithms, such as MPEDE [86], LSHADE [87], ALCPSO [88], CLPSO [38], BMWOA [90], CESCA [89], IGWO [39] and OBLGWO [91], in order to fully prove the ability of the proposed algorithm to avoid local optimal and global exploration. These include two superior DE variants, two often-computed PSO variants, and variants of WOA, GWO, and SCA.

Table A8, Table A9 and Table A10 record the results of the comparison between ISMA and eight advanced algorithms. As can be seen from the comparison results in Table A10, among ISMA and 8 other advanced meta-heuristic algorithms, the average Freidman test result of ISMA is 3.7075758, ranking first, followed by CLPSO. The statistical results in Table A8 show that among all the comparison algorithms, the std of ISMA on more test functions is 0, so it can be seen that ISMA is more stable. In addition, the comparison results of specific functions show that ISMA has a stronger ability to deal with complex functions and mixed functions than other advanced algorithms. The mean and std in Table A8 also indicate the superiority of ISMA in F1–6, F9–15, F26–28 and F30–33 functions. ISMA also ranks high in the F7, F21–23. In addition, Table A9 shows the Wilcoxon signed-rank test results between ISMA and other advanced algorithms. It can be seen that ISMA outperforms other algorithms on most of the benchmark functions, especially CESCA, which outperforms CESCA on 90.9% of the functions. As a result, ISMA is superior to other strong competitors.

**Figure 4:**
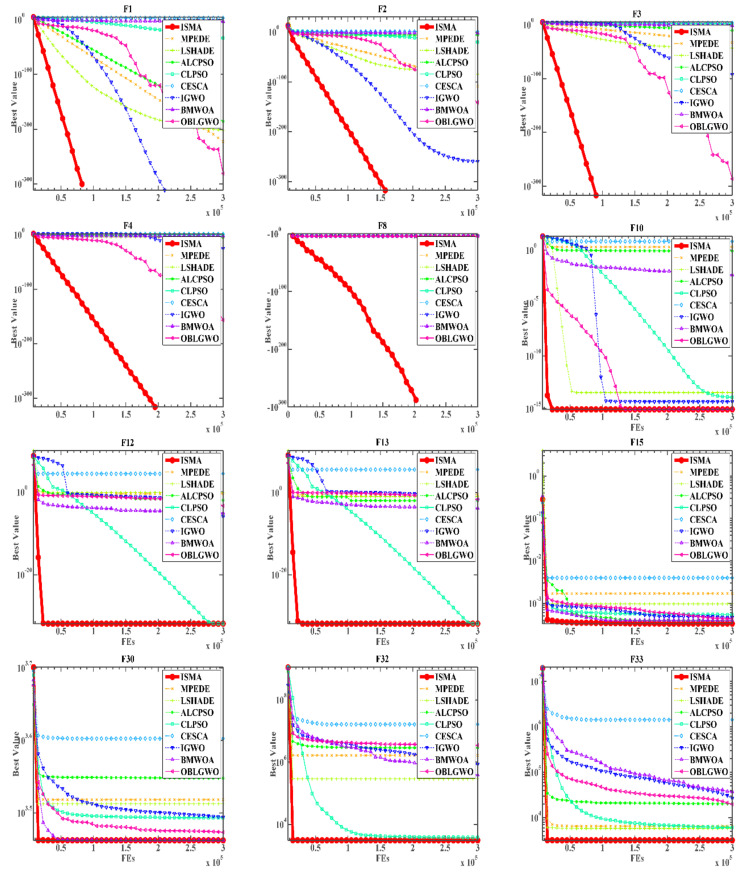
Convergence curves of the ISMA and the other advanced algorithms on twelve functions.

The convergence curves of all nine algorithms over 12 functions shown in Figure 4 show that the convergence rate of ISMA is competitive with other more advanced methods, which always converge to local optimum earlier than ISMA. It can be proved that the ISMA algorithm has a strong ability to avoid local and global searches, and ISMA can produce more accurate solutions.

To sum up, the optimization power of ISMA is reflected in the overall superior performance of ISMA in different types of functions compared to the more challenging advanced methods. The combination of the Cauchy mutation mechanism and crossover mutation strategy based on the DE algorithm enables the proposed ISMA to obtain a higher quality solution in the optimization process and makes exploration and exploitation in a better equilibrium state.

## 5. The Proposed Technique for Gene Selection

In this section, the proposed ISMA is applied to the gene selection problem, which makes improving the proposed algorithm more practical. For this purpose, we transform the continuous ISMA into a discrete version, namely the BISMA of the wrapper method, to solve the gene selection problem for binary optimization tasks.

### 5.1. System Architecture of Gene Selection Based on ISMA

The procedure of selecting or generating some of the most significant features from a set of features in order to lower the dimension of the training dataset is known as feature selection. Many fields with large data sets want to be able to reduce the dimensions of application data, such as gene selection for high-dimensional gene expression data sets in the medical field. The task of gene selection is to reduce the number of irrelevant and unimportant genes, identify the most relevant genomes with the greatest classification accuracy, reduce the cost of high computing costs and improve the accuracy of disease analysis. The continuous ISMA optimizer is converted to binary ISMA (BISMA) using the transfer function (TF) for the gene selection problem. The machine learning algorithm was used as a classifier to evaluate the ability of BISMA to identify discriminant genes and eliminate irrelevant, redundant genes in high-dimensional gene expression datasets. In addition, cross-validation (CV) was used to evaluate the optimality of selected gene subsets for classification during the evaluation process.

### 5.2. Fitness Function

Gene selection is a process that uses the least subset of genes to obtain the optimal classification accuracy, and both goals need to be achieved simultaneously. Therefore, in order to meet each objective, the fitness function expressed in Equation (11) can be designed to comprehensively evaluate the candidate solutions by using classification accuracy and the number of selected genes.

(11) $$fit=\alpha \times \left(1-Acc\right)+\beta \;\times \left(\frac{D_{R}}{D}\right)$$

where *Acc* indicates the classification accuracy of the classifier (machine learning method), so $\left(1-Acc\right)$ is the error rate of the classifier. The weighting factors α and *β* are the importance of error rate and the number of selected genes, respectively, and α ∈ [0,1], *β* = 1 − α. D is the total number of genes in the exponential data set, and the numerator $D_{R}$ is the number of genes filtered by the proposed gene selection optimizer. In this study, α and *β* were set to 0.95 and 0.05, respectively.

### 5.3. Implementation of Discrete BSSMA

The proposed ISMA optimizer searches for the optimal solution in a continuous search space in previous work. Gene selection is a binary problem. The transfer function restricts the continuous search space to 0 or 1. When the value is 0, it means not selected, and when the value is 1, it means selected.

Individuals with binary position vectors are initialized through a random threshold, as shown below:

(12) $$x_{i}^{d}=\left\{\begin{array}{c} 0,\;rand\leq 0.5\\ 1,\;rand>0.5\; \end{array}\right.$$

where $x_{i}^{d}$ is the  i-th gene on the d-th dimension of the position vectors of the slime mould.

In addition, the transfer function (TF) is a suitable converter that can convert a continuous optimization algorithm to a discrete version without changing the algorithm’s structure because it is convenient and efficient [99]. There are 8 types of TFs, which can be divided into S-shaped and V-shaped according to their shapes. Their mathematical formulae and graphical descriptions are shown in Table A11.

For an S-shaped family, a gene of the position vector at the next iteration can be converted according to the TFS1-TFS4 shown in Table A11 as follows:

(13) $$x_{i}^{d}\left(t+1\right)=\left\{\begin{array}{c} 1,\;rand<T\left(x_{i}^{d}\left(t+1\right)\right)\\ 0,\;rand\geq T\left(x_{i}^{d}\left(t+1\right)\right) \end{array}\right.$$

where $T\left(x_{i}^{d}\left(t+1\right)\right)$ represents the probability value of the i-th gene on the d-th dimension at the next iteration.

For a V-shaped family, the gene of the position vector at the next iteration can be converted according to the TFV1-TFV4 shown in Table A11 as follows:

(14) $$x_{i}^{d}\left(t+1\right)=\left\{\begin{array}{c} \neg x_{i}^{d}\left(t+1\right),\;rand<T\left(x_{i}^{d}\left(t+1\right)\right)\\ x_{i}^{d}\left(t+1\right),\;rand\geq T\left(x_{i}^{d}\left(t+1\right)\right) \end{array}\right.$$

## 6. Experimental Design and Discussion on Gene Selection

### 6.1. Experimental Design

In this experiment, two kinds of comparison results are used to evaluate the optimization ability of the proposed algorithm. In the first assessment, we studied BISMA with different TFs to determine the best version of BISMA out of the eight TFs. The resulting BISMA is compared with other mature meta-heuristic optimizers in the second evaluation. Fourteen gene expression datasets were used in the two case studies. Table A12 lists the detailed characteristics of these microarray datasets, including the number of samples, the number of genes per sample, and the number of categories. These 14 representative gene datasets have been widely used to test a variety of gene selection optimizers to evaluate their performance.

In addition, to obtain more convincing results, this paper also considers the Leave-One-Out cross-validation (LOOCV) to validate the gene selection process. A sample in the data set is taken as the test set to verify the classification accuracy of the classifier, while the rest of the sample is taken as the training set to be trained with the classifier. The number of validations per dataset is equal to the size of the test dataset. The KNN classifier is used for classification tasks. For KNN, let the field size k in KNN be 1. The test method of distance D is as follows:

(15) $$D\left(x,y\right)={\left(\sum_{K}^{N}\left(x_{k}-y_{k}\right)\right)}^{\frac{1}{2}}$$

To be fair in comparison [100,101,102], each evaluation and comparison involving BISMA was performed on the same computing environment, namely Intel(R) Xeon(R) Silver 4110 CPU @ 2.40 GHz 2.10 GHz (two processors) and 8 GB RAM (Windows 10)(64-bit). MATLAB R2014a software was used to test the algorithm. For each algorithm, we set the maximum number of iteration agents and the number of search agents to 50 and 20, respectively. It was run 10 times independently. The initial parameters of all algorithms are identified as their original reference parameters.

### 6.2. The Proposed BISMA with Different TFs

Considering the effect of TF on the performance of the gene selection optimizer, we developed eight BISMA optimizers using eight different TFs and evaluated their effectiveness in finding the optimal gene from each gene dataset listed in Table A10. These eight TFs include four S-shaped and four V-shaped TFs, as shown in Table A11. This assessment helps to obtain the best binary version of BISMA to the gene selection issue. Table A13, Table A14, Table A15 and Table A16 show the average number of selected genes, the average error rate, the average fitness, the average calculation time, and the corresponding std and AVR of the 8 developed versions of the BISMA optimizer.

The average number of selected genes produced by each version of BISMA on the 14 datasets is shown in Table A13. The number of genes required by BISMA based on V-shaped was the least among all versions of BISMA. As can be seen from the ARV value, the average number of selected genes of BISMA based on TFV4 was the least and ranked the first, while the four BISMA based on V-shaped were ranked as the first four, and the number of selected genes was significantly lower than that of BISMA based on S-shaped.

Table A14 records the average classification error rates of the eight versions of BISMA on the baseline gene dataset. Judging from the average ranking value, BISMA with TFV4 is significantly better than other competitors. The four V-shaped BISMAs obtained an average error of 0 in 57% of the gene data sets, indicating the stability of feature selection based on V-shaped BISMA. Meanwhile, BISMA based on V4 obtained an error of 0 and a standard deviation of 0 on 85.7% of gene data sets. Therefore, from the average error rate, the ability of BIMSA with V-shaped TFs to solve the gene selection task is due to its S-shaped TFs counterpart.

According to the average fitness test results reported in Table A15, it can be found that BISMA_V3 achieved the best fitness on about 42.9% of the baseline gene data set, which was slightly better than BISMA_V3 and significantly better than other competitors. However, from the ranking mean, BISMA_V4 ranked first, followed by BISMA_V3, BISMA_V1, BISMA_V2, BISMA_V1, BISMA_S2, BISMA_S3, and BISMA_S4. The fitness results also showed that TFs of the V-shaped family were better than that of the S-shaped family.

Similarly, it can be seen from the calculated time that, except for V1, the version of the V-shaped TFs takes less time to run than the version of the S-shaped TFs. In particular, the first-place V4 takes much less time on average than the second-place V3. The calculation overhead of BISMA_V4 with the best average ranking value is lower than that of the other versions over all the benchmark datasets.

As shown in Table A13, Table A14, Table A15 and Table A16, the BISMA version with TFV4 was superior to other versions in terms of the average number of selected genes, average error rate, average fitness, and average time cost, and the BISMA version with TFV4 was far superior to the second in terms of average time cost. In comparing S-shaped and V-shaped, V-shaped can achieve better results than S-shaped. Therefore, the transfer function TFV4 was chosen as the best choice to establish a BISMA optimizer with better stability for genetic problems. In this case, BISMA_V4 is used to represent BISMA, which is further evaluated by comparison in the following sections.

### 6.3. Comparative Evaluation with Other Optimizers

In this section, the superiority of the proposed BISMA optimizer is evaluated by comparing it with several state-of-the-art meta-heuristic approaches. These algorithms considered to be meta-heuristics are bGWO [103], BGSA [104], BPSO [99], bALO [105], BBA [106], BSSA [107], bWOA [108], BSMA, the binary form of the original SMA [35], and BISMA, the discrete version of the improved ISMA. Table A17 shows the parameter settings for the relative comparison optimizer.

Table A18, Table A19, Table A20 and Table A21 show the selected genes’ statistical results in terms of length, error rate, fitness and computational time. According to the average gene length in Table A18, the proposed BISMA had the least number of selected genes on 57.1% of the gene datasets, while bWOA had the least number of selected genes on 42.9% of the gene datasets. It can be seen that in the 14 data sets, BISMA and bWOA are far more competitive than other algorithms in reducing the data dimensions.

The results of the mean error rate are shown in Table A19, which shows the superiority of the proposed BISMA. BISMA achieves the minimum mean error rate on 85.7% of the gene data sets and only performs slightly worse on Lung_Cancer and Tumor_14. bGWO showed the best error rate on the Tumor_14 gene dataset, while bWOA showed competing results on the Lung_Cancer gene dataset. From the perspective of the ARV index, BISMA ranked first, followed by bWOA, bGWO, BISMA, BGSA, BPSO, bALO, BSSA, and BBA.

The fitness of the important measurements shown in Table A20 comprises the weighted error rate and the number of genes selected by weighting. It is clear that the performance of the proposed BISMA is superior to other competitors on 64.3% of the gene data sets. The average fitness of BISMA and bWOA on 14 gene datasets was significantly better than that of the other algorithms.

In addition, according to the std values shown in Table A18, Table A19 and Table A20, BISMA showed better performance, satisfactory standard deviation and excellent average fitness values in most of the gene data sets tested, which indicated that BISMA was more stable than bALO, BSSA, BBA, etc. There is a big gap between the overall performance of BISMA, BSMA, bWOA, and bGWO and the overall performance of BGSA, BPSO, bALO, BBA, and BSSA, and the first four optimizers are obviously better than the last five.

As can be seen from the average calculation time results shown in Table A21, the proposed BISMA has the highest time cost, and the time complexity of BSMA and bWOA with better performance is also relatively high, indicating the increase in calculation time cost caused by the improvement of performance. The time cost of BISMA was influenced by the introduced Cauchy mutation and the crossover mutation strategy based on DE. As shown in Table A21, the calculation time of the original SMA is also relatively expensive, which is also the reason for the high cost of BISMA time.

Compared with other gene selection optimizers, it is found that BISMA is the best one. Although the result is not ideal in terms of calculation time, BISMA is expected to select the optimal gene subset on the vast majority of microarray data sets to obtain the best fitness and the best classification error rate without the loss of meaningful genes. This fact proves that the combination of Cauchy mutation and crossover mutation strategy based on DE guarantees the improvement of global exploration in the proposed BISMA to achieve a more effective balance between local exploitation and global exploration.

## 7. Discussions

In this part, the ISMA algorithm proposed in this paper is discussed, and its advantages and existing points can be improved. In the original SMA, the global exploration ability of slime moulds was not strong, and they would fall into local optimum in the face of some problems, limiting the algorithm’s use. In this paper, Cauchy mutation (CM) and cross mutation are introduced to update the population, increasing the global exploration space and avoiding falling into local optimum. Experiments show that the effect of a dual mechanism is better than that of a single mechanism, and ISMA is better than some advanced optimization algorithms.

However, ISMA exposes some common shortcomings of random optimizers in certain areas. As seen in Table A5 and Table A8, when processing some multimodal functions, the algorithm’s performance is sometimes poor due to the randomness of the crossover mutation mechanism. The search speed is slow in global exploration and local exploitation.

A binary algorithm (BISMA) performs feature selection in feature selection optimization problems on 14 data sets. The experimental results show that the proposed algorithm exhibits smaller average fitness and lower classification error rates while selecting fewer features. However, the introduction of Cauchy mutation and cross mutation mechanism brings good effects but also leads to a long running time of the algorithm, and the time complexity is the highest among all comparison algorithms.

According to the study [109], Ornek et al. combined the position update of the sines and cosines algorithm with the slime mold algorithm. In these updates, various sines and cosines algorithms are used to modify the oscillation process of slime molds. Experimental results show that the algorithm has good exploration and exploitation ability. Gurses et al. [110] applied a new hybrid slime mold algorithm, the Simulated Annealing Algorithm (HSMA-SA), to structural engineering design problems. Experimental results demonstrate the feasibility of the proposed algorithm in solving shape optimization problems. Cai et al. [111] proposed an artificial slime mold algorithm to solve the traffic network node selection problem, and the experimental results are of great significance to studying traffic node selection and artificial learning mechanisms. These ideas can be used as a reference to improve the shortcomings of ISMA in the future so that it can be applied in more fields, such as dynamic module detection [112,113], road network planning [114], information retrieval services [115,116,117], drug discovery [118,119], microgrids planning [120], image dehazing [121], location-based services [122,123], power flow optimization [124], disease identification and diagnosis [125,126], recommender system [127,128,129,130], human activity recognition [131], and image-to-image translation [132].

## 8. Conclusions

In this study, based on the basic SMA, an improved ISMA version is proposed, and the combination of the Cauchy mutation and crossover mutation strategy based on the DE algorithm is used to improve the SMA so as to achieve the coordination between global exploration and local exploitation. We first evaluate the effectiveness of the continuous version of the ISMA algorithm on 33 benchmark evaluation functions to deal with global optimization problems, compared with some advanced swarm intelligence algorithms. The results show that ISMA has a strong global exploration capability. In order to verify the performance of ISMA in practical application, the BISMA was obtained by mapping ISMA into binary space through the transfer function and then applied to the feature selection problem of 14 commonly used UCI datasets. In order to understand the optimal conversion function of the ISMA variant, we compared the number of selected genes, average error rate, average fitness, and computational cost. It can be seen that BISMA_V4 is superior to other versions. Therefore, BISMA_V4 is regarded as the final method to solve the gene selection problem. We compare BISMA_V4 with binary SMA, binary GWO, and several other advanced methods. The experimental results show that BISMA can select fewer features and obtain higher classification accuracy.

Therefore, we believe that the proposed BISMA is a promising gene selection technique. There are several ways to extend the work we have conducted. We can consider applying BISMA to other high-dimensional data sets and study the effectiveness of BISMA on other data sets. Secondly, other strategies can be used to improve the SMA and improve the coordination between the SMA global exploration and local exploration. Thirdly, interested researchers can apply SMA to more areas, such as financial forecasting, optimization of photovoltaic parameters, and other engineering applications. Finally, we can extend the application of ISMA to multi-objective optimization, image segmentation, machine learning, and other fields.

## Data Availability

The data involved in this study are all public data, which can be downloaded through public channels.

## Appendix A

See Table A1, Table A2, Table A3, Table A4, Table A5, Table A6, Table A7, Table A8, Table A9, Table A10, Table A11, Table A12, Table A13, Table A14, Table A15, Table A16, Table A17, Table A18, Table A19, Table A20 and Table A21.

**Table A1:** Descriptions of unimodal benchmark functions.

Function	*Dim*	*Range*	*f*_min_
$f_{1}\left(x\right)=\sum_{i=1}^{n}x_{i}^{2}$	*30*	[−100, 100]	0
$f_{2}\left(x\right)=\sum_{i=1}^{n}\left|\;x_{i}\right|$ + $\Pi_{i=1}^{n}\left|x_{i}\right|$	*30*	[−10, 10]	0
$f_{3}\left(x\right)=\sum_{i=1}^{n}{\left(\Sigma_{j-1}^{i}x_{j}\right)}^{2}$	*30*	[−100, 100]	0
$f_{4}\left(x\right)=max_{i}${$\left|\;x_{i}\right|,\;1\;\leq \;i\;\leq n\}$	*30*	[−100, 100]	0
$f_{5}\left(x\right)=\sum_{i=1}^{n-1}[100{\left(x_{i+1}-x_{i}^{2}\right)}^{2}$ + ${\left(x_{i}-1\right)}^{2}$]	*30*	[−30, 30]	0
$f_{6}\left(x\right)=\sum_{i=1}^{n}{\left(\left[x_{i}+0.5\right]\right)}^{2}$	*30*	[−100, 100]	0
$f_{7}\left(x\right)=\sum_{i=1}^{n}ix_{i}^{4}$ + *random*[0,1)	*30*	[−128, 128]	0

**Table A2:** Descriptions of multimodal benchmark functions.

Function	*Dim*	*Range*	*f*_min_
$f_{8}\left(x\right)\;=\sum_{i=1}^{n}$ − $x_{i}sin\left(\sqrt{\left|x_{i}\right|}\;\right)$	*30*	[−500, 500]	−418.9829 × 30
$f_{9}\left(x\right)\;=\sum_{i=1}^{n}[x_{i}^{2}-\;10$*cos*(2$\pi x_{i})$ + 10]	*30*	[−5.12, 5.12]	0
$f_{10}\left(x\right)\;=$ −20 *exp*{−0.2$\sqrt{\frac{1}{n}\Sigma_{i=1}^{n}x_{i}}\;\}-exp${$\frac{1}{n}\Sigma_{i=1}^{n}\cos(2\pi x_{i}$)} + 20 + e	*30*	[−32, 32]	0
$f_{11}\left(x\right)=\frac{1}{4000}\Sigma_{i=1}^{n}x_{i}^{2}-\Pi_{i=1}^{n}cos\left(\frac{x_{i}}{\sqrt{i}}\right)+1$	*30*	[−600, 600]	0
$f_{12}\left(x\right)\;=\frac{\pi }{n}${10*sin*($ay_{1}$)+ $\sum_{i=1}^{n-1}{\left(y_{i}-1\right)}^{2}$[1+10$sin^{2}$($\pi y_{i+1}$)]+ ${\left(y_{n}-1\right)}^{2}$+$\sum_{i=1}^{n}\mu \left(x_{i},10,100,4\right)\}$ $y_{i}=1+\frac{x_{i}+1}{4}$ $\mu \left(x_{i},a,k,m\right)=\left\{\begin{array}{c} k{\left(x_{i}-a\right)}^{m}\;x_{i}>a\\ 0\;\;-a<x_{i}<a\\ k{\left(-x_{i}-a\right)}^{m}\;x\;<-a \end{array}\right.$	*30*	[−50, 50]	0
$f_{13}\left(x\right)\;=0.01\{sin^{2}$(3$\pi x_{i}$)+$\sum_{i=1}^{n}{\left(x_{i}-1\right)}^{2}$[1+$sin^{2}$(3$\pi x_{i}+1$)]+ ${\left(x_{n}-1\right)}^{2}$[1+$sin^{2}$(2$\pi x_{n}$)]+ $\sum_{i=1}^{n}\;\mu \left(x_{i},5,100,4\right)$	*30*	[−50, 50]	0

**Table A3:** Descriptions of fixed-dimension multimodal benchmark functions.

Function	*Dim*	*Range*	*f*_min_
$f_{14}\left(x\right)={\left(\frac{1}{500}+\Sigma_{j=1}^{25}\frac{1}{j+\Sigma_{j=1}^{2}{\left(x_{i}-a_{ij}\right)}^{6}}\right)}^{-1}$	2	[−65, 65]	1
$f_{15}\left(x\right)=\Sigma_{i=1}^{11}{\left[a_{i}-\frac{x_{1}\left(b_{i}^{2}+b_{i}x_{2}\right)}{b_{i}^{2}+b_{i}x_{3}+x_{4}}\right]}^{2}$	4	[−5, 5]	0.00030
$f_{16}\left(x\right)=4x_{1}^{2}-2.1x_{i}^{4}+\frac{1}{3}x_{1}^{6}+x_{1}x_{2}-4x_{2}^{2}+4x_{2}^{4}$	2	[−5, 5]	−1.0316
$f_{17}\left(x\right)\;={\left(x_{2}-\frac{5.1}{4\pi^{2}}x_{1}^{2}+\frac{5}{\pi }x_{1}-6\right)}^{2}$ + 10(1 − $\frac{1}{8\pi }$)cos $x_{1}+10$	2	[−5, 5]	0.398
$f_{18}\left(x\right)\;=$ [1 + ${\left(x_{1}+x_{2}+1\right)}^{2}$(19 − 14$x_{1}+3x_{1}^{2}-14x_{2}+6x_{1}x_{2}$ + 3$x_{2}^{2}$)] × [30 + ${\left(2x_{1}-3x_{2}\right)}^{2}$×(18 − 32$x_{1}+12x_{1}^{2}$ + 48$x_{2}$ − 36$x_{1}x_{2}$ + 27$x_{2}^{2})]$	2	[−2, 2]	3
$f_{19}\left(x\right)=-\sum_{i=1}^{4}c_{i}\exp\left(-\sum_{j=1}^{3}a_{ij}{\left(x_{j}-P_{ij}\right)}^{2}\right)$	3	[1, 3]	−3.86
$f_{20}\left(x\right)=-\sum_{i=1}^{4}c_{i}\exp\left(-\sum_{j=1}^{6}a_{ij}{\left(x_{j}-P_{ij}\right)}^{2}\right)$	6	[0, 1]	−3.32
$f_{21}\left(x\right)=-\sum_{i=1}^{5}{\left[\left(X-a_{i}\right){\left(X-a_{i}\right)}^{T}+c_{i}\right]}^{-1}$	4	[0, 10]	−10.1532
$f_{22}\left(x\right)=-\sum_{i=1}^{7}{\left[\left(X-a_{i}\right){\left(X-a_{i}\right)}^{T}+c_{i}\right]}^{-1}$	4	[0, 10]	−10.4028
$f_{23}\left(x\right)=-\sum_{i=1}^{10}{\left[\left(X-a_{i}\right){\left(X-a_{i}\right)}^{T}+c_{i}\right]}^{-1}$	4	[0, 10]	−10.5363

**Table A4:** Descriptions of CEC2014 functions. (Search range: [−100, 100]D).

Function	Class	Functions	Optimum
F24	Hybrid	Hybrid Function 5 (N = 5)	2100
F25		Hybrid Function 6 (N = 5)	2200
F26	Composition	Composition Function 1 (N = 5)	2300
F27		Composition Function 2 (N = 3)	2400
F28		Composition Function 3 (N = 3)	2500
F29		Composition Function 4 (N = 5)	2600
F30		Composition Function 5 (N = 5)	2700
F31		Composition Function 6 (N = 5)	2800
F32		Composition Function 7 (N = 3)	2900
F33		Composition Function 8 (N = 3)	3000

**Table A5:** The SMA variants are compared with the original SMA and DE algorithms.

	F1		F2		F3	
	mean	std	mean	mean	std	mean
ISMA	**0.0000 × 10^0^**	**0.0000 × 10^0^**	**0.0000 × 10^0^**	**0.0000 × 10^0^**	**0.0000 × 10^0^**	**0.0000 × 10^0^**
SMA	3.2559 × 10^−44^	1.7833 × 10^−43^	1.7856 × 10^−44^	3.2559 × 10^−44^	1.7833 × 10^−43^	1.7856 × 10^−44^
CSMA	0.0000 × 10^0^	0.0000 × 10^0^	0.0000 × 10^0^	0.0000 × 10^0^	0.0000 × 10^0^	0.0000 × 10^0^
MCSMA	0.0000 × 10^0^	0.0000 × 10^0^	0.0000 × 10^0^	0.0000 × 10^0^	0.0000 × 10^0^	0.0000 × 10^0^
DE	1.8673 × 10^−159^	4.1198 × 10^−159^	1.3001 × 10^−94^	1.8673 × 10^−159^	4.1198 × 10^−159^	1.3001 × 10^−94^
	F4		F5		F6	
	mean	std	mean	mean	std	mean
ISMA	**0.0000 × 10^0^**	**0.0000 × 10^0^**	**1.5210 × 10^−20^**	**0.0000 × 10^0^**	**0.0000 × 10^0^**	**1.5210 × 10^−20^**
SMA	9.1947 × 10^−44^	5.0362 × 10^−43^	4.5273 × 10^−1^	9.1947 × 10^−44^	5.0362 × 10^−43^	4.5273 × 10^−1^
CSMA	0.0000 × 10^0^	0.0000 × 10^0^	1.0735 × 10^0^	0.0000 × 10^0^	0.0000 × 10^0^	1.0735 × 10^0^
MCSMA	5.5509 × 10^−247^	0.0000 × 10^0^	3.7675 × 10^0^	5.5509 × 10^−247^	0.0000 × 10^0^	3.7675 × 10^0^
DE	6.3804 × 10^−15^	1.3750 × 10^−14^	3.2827 × 10^1^	6.3804 × 10^−15^	1.3750 × 10^−14^	3.2827 × 10^1^
	F7		F8		F9	
	mean	std	mean	mean	std	mean
ISMA	5.2004 × 10^−5^	4.4680 × 10^−5^	6.5535 × 10^4^	5.2004 × 10^−5^	4.4680 × 10^−5^	6.5535 × 10^4^
SMA	1.8109 × 10^−3^	1.9112 × 10^−3^	−1.256 × 10^4^	1.8109 × 10^−3^	1.9112 × 10^−3^	−1.256 × 10^4^
CSMA	**1.0466 × 10^−5^**	**7.1026 × 10^−6^**	6.5535 × 10^4^	**1.0466 × 10^−5^**	**7.1026 × 10^−6^**	6.5535 × 10^4^
MCSMA	2.8153 × 10^−4^	1.4821 × 10^−4^	**−** **1.256 × 10^4^**	2.8153 × 10^−4^	1.4821 × 10^−4^	**−** **1.256 × 10^4^**
DE	2.4715 × 10^−3^	4.9474 × 10^−4^	−1.244 × 10^4^	2.4715 × 10^−3^	4.9474 × 10^−4^	−1.244 × 10^4^
	F10		F11		F12	
	mean	std	mean	mean	std	mean
ISMA	**8.8818 × 10^−16^**	**0.0000 × 10^0^**	**0.0000 × 10^0^**	**8.8818 × 10^−16^**	**0.0000 × 10^0^**	**0.0000 × 10^0^**
SMA	8.8818 × 10^−16^	0.0000 × 10^0^	0.0000 × 10^0^	8.8818 × 10^−16^	0.0000 × 10^0^	0.0000 × 10^0^
CSMA	8.8818 × 10^−16^	0.0000 × 10^0^	0.0000 × 10^0^	8.8818 × 10^−16^	0.0000 × 10^0^	0.0000 × 10^0^
MCSMA	8.8818 × 10^−16^	0.0000 × 10^0^	0.0000 × 10^0^	8.8818 × 10^−16^	0.0000 × 10^0^	0.0000 × 10^0^
DE	7.7568 × 10^−15^	9.0135 × 10^−16^	0.0000 × 10^0^	7.7568 × 10^−15^	9.0135 × 10^−16^	0.0000 × 10^0^
	F13		F14		F15	
	mean	std	mean	mean	std	mean
ISMA	**1.3498 × 10^−32^**	**5.5674 × 10^−48^**	**9.9800 × 10^−1^**	**1.3498 × 10^−32^**	**5.5674 × 10^−48^**	**9.9800 × 10^−1^**
SMA	4.8249 × 10^−3^	7.4218 × 10^−3^	1.3350 × 10^0^	4.8249 × 10^−3^	7.4218 × 10^−3^	1.3350 × 10^0^
CSMA	4.3078 × 10^−3^	6.3340 × 10^−3^	1.2955 × 10^0^	4.3078 × 10^−3^	6.3340 × 10^−3^	1.2955 × 10^0^
MCSMA	1.3498 × 10^−32^	5.5674 × 10^−48^	9.9800 × 10^−1^	1.3498 × 10^−32^	5.5674 × 10^−48^	9.9800 × 10^−1^
DE	1.3498 × 10^−32^	5.5674 × 10^−48^	1.0311 × 10^0^	1.3498 × 10^−32^	5.5674 × 10^−48^	1.0311 × 10^0^
	F16		F17		F18	
	mean	std	mean	mean	std	mean
ISMA	−1.032 × 10^0^	1.2770 × 10^−8^	3.9838 × 10^−1^	−1.032 × 10^0^	1.2770 × 10^−8^	3.9838 × 10^−1^
SMA	−8.2436 × 10^−1^	4.1923 × 10^−1^	4.1640 × 10^−1^	−8.2436 × 10^−1^	4.1923 × 10^−1^	4.1640 × 10^−1^
CSMA	−1.031 × 10^0^	1.1109 × 10^−3^	4.1829 × 10^−1^	−1.031 × 10^0^	1.1109 × 10^−3^	4.1829 × 10^−1^
MCSMA	−1.031 × 10^0^	6.5572 × 10^−4^	3.9865 × 10^−1^	−1.031 × 10^0^	6.5572 × 10^−4^	3.9865 × 10^−1^
DE	**−1.031 × 10^0^**	**6.7752 × 10^−16^**	**3.9789 × 10^−1^**	**−1.031 × 10^0^**	**6.7752 × 10^−16^**	**3.9789 × 10^−1^**
	F19		F20		F21	
	mean	std	mean	mean	std	mean
ISMA	−3.863 × 10^0^	1.1037 × 10^−4^	−3.163 × 10^0^	−3.863 × 10^0^	1.1037 × 10^−4^	−3.163 × 10^0^
SMA	−3.782 × 10^0^	9.4398 × 10^−2^	−2.958 × 10^0^	−3.782 × 10^0^	9.4398 × 10^−2^	−2.958 × 10^0^
CSMA	−3.795 × 10^0^	7.9965 × 10^−2^	−2.901 × 10^0^	−3.795 × 10^0^	7.9965 × 10^−2^	−2.901 × 10^0^
MCSMA	−3.861 × 10^0^	1.9880 × 10^−3^	−3.042 × 10^0^	−3.861 × 10^0^	1.9880 × 10^−3^	−3.042 × 10^0^
DE	**−** **3.862 × 10^0^**	**2.7101 × 10^−15^**	**−3** **.321 × 10^0^**	**−** **3.862 × 10^0^**	**2.7101 × 10^−15^**	**−3** **.321 × 10^0^**
	F22		F23		F24	
	mean	std	mean	mean	std	mean
ISMA	−1.040 × 10^1^	3.3560 × 10^−6^	^−1.0^54 × 10^1^	−1.040 × 10^1^	3.3560 × 10^−6^	^−1.0^54 × 10^1^
SMA	−1.032 × 10^1^	9.7684 × 10^−2^	−1.044 × 10^1^	−1.032 × 10^1^	9.7684 × 10^−2^	−1.044 × 10^1^
CSMA	−9.877 × 10^0^	1.2268 × 10^0^	−1.041 × 10^1^	−9.877 × 10^0^	1.2268 × 10^0^	−1.041 × 10^1^
MCSMA	−1.040 × 10^1^	6.2358 × 10^−6^	−1.054 × 10^1^	−1.040 × 10^1^	6.2358 × 10^−6^	−1.054 × 10^1^
DE	**−1.040 × 10^1^**	**1.8067 × 10^−15^**	**−1.053 × 10^1^**	**−1.040 × 10^1^**	**1.8067 × 10^−15^**	**−1.053 × 10^1^**
	F25		F26		F27	
	mean	std	mean	mean	std	mean
ISMA	3.4989 × 10^3^	2.2734 × 10^2^	**2.5000 × 10^3^**	3.4989 × 10^3^	2.2734 × 10^2^	**2.5000 × 10^3^**
SMA	1.0429 × 10^4^	2.8215 × 10^4^	2.5169 × 10^3^	1.0429 × 10^4^	2.8215 × 10^4^	2.5169 × 10^3^
CSMA	4.7397 × 10^3^	1.2900 × 10^3^	2.5000 × 10^3^	4.7397 × 10^3^	1.2900 × 10^3^	2.5000 × 10^3^
MCSMA	3.6251 × 10^3^	1.8988 × 10^2^	2.5000 × 10^3^	3.6251 × 10^3^	1.8988 × 10^2^	2.5000 × 10^3^
DE	**2.3554 × 10^3^**	**8.2085 × 10^1^**	2.6152 × 10^3^	**2.3554 × 10^3^**	**8.2085 × 10^1^**	2.6152 × 10^3^
	F28		F29		F30	
	mean	std	mean	mean	std	mean
ISMA	**2.7000 × 10^3^**	**0.0000 × 10^0^**	2.7147 × 10^3^	**2.7000 × 10^3^**	**0.0000 × 10^0^**	2.7147 × 10^3^
SMA	2.7000 × 10^3^	0.0000 × 10^0^	2.7732 × 10^3^	2.7000 × 10^3^	0.0000 × 10^0^	2.7732 × 10^3^
CSMA	2.7000 × 10^3^	0.0000 × 10^0^	2.7172 × 10^3^	2.7000 × 10^3^	0.0000 × 10^0^	2.7172 × 10^3^
MCSMA	2.7000 × 10^3^	0.0000 × 10^0^	2.7788 × 10^3^	2.7000 × 10^3^	0.0000 × 10^0^	2.7788 × 10^3^
DE	2.7066 × 10^3^	8.5796 × 10^−1^	**2.7003 × 10^3^**	2.7066 × 10^3^	8.5796 × 10^−1^	**2.7003 × 10^3^**
	F31		F32		F33	
	mean	std	mean	mean	std	mean
ISMA	**3.0000 × 10^3^**	**0.0000 × 10^0^**	**3.1000 × 10^3^**	**3.0000 × 10^3^**	**0.0000 × 10^0^**	**3.1000 × 10^3^**
SMA	4.1186 × 10^3^	1.9606 × 10^3^	2.8989 × 10^7^	4.1186 × 10^3^	1.9606 × 10^3^	2.8989 × 10^7^
CSMA	3.0000 × 10^3^	0.0000 × 10^0^	3.1000 × 10^3^	3.0000 × 10^3^	0.0000 × 10^0^	3.1000 × 10^3^
MCSMA	5.4386 × 10^3^	1.1178 × 10^3^	4.0742 × 10^7^	5.4386 × 10^3^	1.1178 × 10^3^	4.0742 × 10^7^
DE	3.6286 × 10^3^	2.4807 × 10^1^	1.2080 × 10^5^	3.6286 × 10^3^	2.4807 × 10^1^	1.2080 × 10^5^

**Table A6:** Wilcoxon signed-rank test results between the SMA variants and the original SMA and DE algorithms.

Function	SMA	CSMA	MCSMA	DE
F1	1.7344 × 10^−6^	1.0000 × 10^0^	1.0000 × 10^0^	1.7344 × 10^−6^
F2	1.7344 × 10^−6^	1.0000 × 10^0^	1.0000 × 10^0^	1.7344 × 10^−6^
F3	1.7344 × 10^−6^	1.0000 × 10^0^	1.0000 × 10^0^	1.7344 × 10^−6^
F4	1.7344 × 10^−6^	1.0000 × 10^0^	1.7344 × 10^−6^	1.7344 × 10^−6^
F5	1.7344 × 10^−6^	1.7344 × 10^−6^	2.3438 × 10^−2^	1.7344 × 10^−6^
F6	1.7344 × 10^−6^	1.7344 × 10^−6^	1.0000 × 10^0^	1.0000 × 10^0^
F7	2.3534 × 10^−6^	4.0715 × 10^−5^	2.6033 × 10^−6^	1.7344 × 10^−6^
F8	1.6503 × 10^−1^	1.2720 × 10^−1^	1.3851 × 10^−1^	1.6268 × 10^−1^
F9	1.0000 × 10^0^	1.0000 × 10^0^	1.0000 × 10^0^	5.0000 × 10^−1^
F10	1.0000 × 10^0^	1.0000 × 10^0^	1.0000 × 10^0^	1.0135 × 10^−7^
F11	1.0000 × 10^0^	1.0000 × 10^0^	1.0000 × 10^0^	1.0000 × 10^0^
F12	1.7344 × 10^−6^	1.7344 × 10^−6^	1.0000 × 10^0^	1.0000 × 10^0^
F13	1.7344 × 10^−6^	1.7344 × 10^−6^	1.0000 × 10^0^	1.0000 × 10^0^
F14	1.7344 × 10^−6^	1.7344 × 10^−6^	1.0000 × 10^0^	1.0000 × 10^0^
F15	2.8786 × 10^−6^	2.6033 × 10^−6^	6.7328 × 10^−1^	3.5888 × 10^−4^
F16	1.7344 × 10^−6^	1.7344 × 10^−6^	1.7344 × 10^−6^	1.7344 × 10^−6^
F17	1.2381 × 10^−5^	8.4661 × 10^−6^	9.5899 × 10^−1^	1.7344 × 10^−6^
F18	7.3433 × 10^−1^	4.0483 × 10^−1^	1.1973 × 10^−3^	1.7344 × 10^−6^
F19	1.7344 × 10^−6^	1.7344 × 10^−6^	2.6033 × 10^−6^	1.7344 × 10^−6^
F20	6.3391 × 10^−6^	6.3391 × 10^−6^	2.6033 × 10^−6^	1.7344 × 10^−6^
F21	1.7344 × 10^−6^	1.7344 × 10^−6^	9.0993 × 10^−1^	3.1123 × 10^−5^
F22	1.7344 × 10^−6^	1.7344 × 10^−6^	1.9569 × 10^−2^	1.7344 × 10^−6^
F23	1.7344 × 10^−6^	1.7344 × 10^−6^	4.2843 × 10^−1^	1.7344 × 10^−6^
F24	6.9838 × 10^−6^	2.5967 × 10^−5^	3.1618 × 10^−3^	1.7344 × 10^−6^
F25	3.1123 × 10^−5^	1.1265 × 10^−5^	4.2767 × 10^−2^	1.7344 × 10^−6^
F26	2.5000 × 10^−1^	1.0000 × 10^0^	1.0000 × 10^0^	4.3205 × 10^−8^
F27	5.0000 × 10^−1^	1.0000 × 10^0^	1.0000 × 10^0^	1.7344 × 10^−6^
F28	1.0000 × 10^0^	1.0000 × 10^0^	1.0000 × 10^0^	1.7344 × 10^−6^
F29	6.5213 × 10^−6^	1.8326 × 10^−3^	1.6789 × 10^−5^	1.7344 × 10^−6^
F30	3.7896 × 10^−6^	1.0000 × 10^0^	1.7344 × 10^−6^	1.7344 × 10^−6^
F31	4.8828 × 10^−4^	1.0000 × 10^0^	2.5631 × 10^−6^	1.7344 × 10^−6^
F32	7.8125 × 10^−3^	1.0000 × 10^0^	1.7344 × 10^−6^	1.7344 × 10^−6^
F33	3.7896 × 10^−6^	1.0000 × 10^0^	1.7344 × 10^−6^	1.7344 × 10^−6^
+/=/−	25/8/0	16/16/1	15/18/1	16/7/10

**Table A7:** Average ranking values using the Friedman test.

Algorithm	ISMA	SMA	CSMA	MCSMA	DE
AVR	2.256060606	3.847979798	3.202525253	2.90959596	2.783838384
rank	1	5	4	3	2

**Table A8:** Comparison of the numerical results obtained by ISMA and other advanced methods.

	F1		F2		F3	
	mean	std	mean	mean	std	mean
ISMA	**0.0000 × 10^0^**	**0.0000 × 10^0^**	**0.0000 × 10^0^**	**0.0000 × 10^0^**	**0.0000 × 10^0^**	**0.0000 × 10^0^**
MPEDE	5.6838 × 10^−223^	0.0000 × 10^0^	2.0352 × 10^−109^	5.6838 × 10^−223^	0.0000 × 10^0^	2.0352 × 10^−109^
LSHADE	8.6954 × 10^−203^	0.0000 × 10^0^	2.6224 × 10^−85^	8.6954 × 10^−203^	0.0000 × 10^0^	2.6224 × 10^−85^
ALCPSO	4.5530 × 10^−186^	0.0000 × 10^0^	1.0128 × 10^−6^	4.5530 × 10^−186^	0.0000 × 10^0^	1.0128 × 10^−6^
CLPSO	2.7917 × 10^−34^	2.0632 × 10^−34^	5.6730 × 10^−21^	2.7917 × 10^−34^	2.0632 × 10^−34^	5.6730 × 10^−21^
CESCA	1.0264 × 10^3^	7.6509 × 10^2^	7.2069 × 10^0^	1.0264 × 10^3^	7.6509 × 10^2^	7.2069 × 10^0^
IGWO	0.0000 × 10^0^	0.0000 × 10^0^	5.4179 × 10^−260^	0.0000 × 10^0^	0.0000 × 10^0^	5.4179 × 10^−260^
BMWOA	8.7826 × 10^−4^	1.9389 × 10^−3^	8.5362 × 10^−3^	8.7826 × 10^−4^	1.9389 × 10^−3^	8.5362 × 10^−3^
OBLGWO	2.6476 × 10^−281^	0.0000 × 10^0^	5.6311 × 10^−142^	2.6476 × 10^−281^	0.0000 × 10^0^	5.6311 × 10^−142^
	F4		F5		F6	
	mean	std	mean	mean	std	mean
ISMA	**0.0000 × 10^0^**	**0.0000 × 10^0^**	**5.6931 × 10^−12^**	**0.0000 × 10^0^**	**0.0000 × 10^0^**	**5.6931 × 10^−12^**
MPEDE	1.3923 × 10^−5^	2.6447 × 10^−5^	1.1960 × 10^0^	1.3923 × 10^−5^	2.6447 × 10^−5^	1.1960 × 10^0^
LSHADE	1.3040 × 10^−4^	2.3249 × 10^−4^	5.3155 × 10^−1^	1.3040 × 10^−4^	2.3249 × 10^−4^	5.3155 × 10^−1^
ALCPSO	2.6029 × 10^−5^	3.4443 × 10^−5^	2.5603 × 10^1^	2.6029 × 10^−5^	3.4443 × 10^−5^	2.5603 × 10^1^
CLPSO	1.3451 × 10^0^	2.6110 × 10^−1^	6.5461 × 10^−1^	1.3451 × 10^0^	2.6110 × 10^−1^	6.5461 × 10^−1^
CESCA	2.0286 × 10^1^	7.5303 × 10^0^	2.4759 × 10^5^	2.0286 × 10^1^	7.5303 × 10^0^	2.4759 × 10^5^
IGWO	7.5149 × 10^−26^	4.1158 × 10^−25^	2.3186 × 10^1^	7.5149 × 10^−26^	4.1158 × 10^−25^	2.3186 × 10^1^
BMWOA	3.6139 × 10^−3^	3.9430 × 10^−3^	3.9781 × 10^−3^	3.6139 × 10^−3^	3.9430 × 10^−3^	3.9781 × 10^−3^
OBLGWO	2.7133 × 10^−157^	1.4861 × 10^−156^	2.6112 × 10^1^	2.7133 × 10^−157^	1.4861 × 10^−156^	2.6112 × 10^1^
	F7		F8		F9	
	mean	std	mean	mean	std	mean
ISMA	9.4873 × 10^−5^	6.6385 × 10^−5^	6.5535 × 10^4^	9.4873 × 10^−5^	6.6385 × 10^−5^	6.5535 × 10^4^
MPEDE	3.2148 × 10^−3^	1.6021 × 10^−3^	−1.187 × 10^4^	3.2148 × 10^−3^	1.6021 × 10^−3^	−1.187 × 10^4^
LSHADE	6.5393 × 10^−3^	5.0546 × 10^−3^	−1.895 × 10^3^	6.5393 × 10^−3^	5.0546 × 10^−3^	−1.895 × 10^3^
ALCPSO	9.6181 × 10^−2^	3.9035 × 10^−2^	−1.147 × 10^4^	9.6181 × 10^−2^	3.9035 × 10^−2^	−1.147 × 10^4^
CLPSO	2.6752 × 10^−3^	7.7407 × 10^−4^	**−1.256 × 10^4^**	2.6752 × 10^−3^	7.7407 × 10^−4^	**−1.256 × 10^4^**
CESCA	5.3895 × 10^−1^	3.4475 × 10^−1^	−3.901 × 10^3^	5.3895 × 10^−1^	3.4475 × 10^−1^	−3.901 × 10^3^
IGWO	2.7827 × 10^−4^	2.2936 × 10^−4^	−7.436 × 10^3^	2.7827 × 10^−4^	2.2936 × 10^−4^	−7.436 × 10^3^
BMWOA	1.1610 × 10^−3^	8.5016 × 10^−4^	−1.257 × 10^4^	1.1610 × 10^−3^	8.5016 × 10^−4^	−1.257 × 10^4^
OBLGWO	**2.3640 × 10^−5^**	**2.4037 × 10^−5^**	−1.253 × 10^4^	**2.3640 × 10^−5^**	**2.4037 × 10^−5^**	−1.253 × 10^4^
	F10		F11		F12	
	mean	std	mean	mean	std	mean
ISMA	**8.8818 × 10^−16^**	**0.0000 × 10^0^**	**0.0000 × 10^0^**	**8.8818 × 10^−16^**	**0.0000 × 10^0^**	**0.0000 × 10^0^**
MPEDE	2.0353 × 10^0^	6.7054 × 10^−1^	1.5065 × 10^−2^	2.0353 × 10^0^	6.7054 × 10^−1^	1.5065 × 10^−2^
LSHADE	3.3455 × 10^−14^	3.7417 × 10^−15^	1.2274 × 10^−2^	3.3455 × 10^−14^	3.7417 × 10^−15^	1.2274 × 10^−2^
ALCPSO	8.3257 × 10^−1^	8.5957 × 10^−1^	1.7674 × 10^−2^	8.3257 × 10^−1^	8.5957 × 10^−1^	1.7674 × 10^−2^
CLPSO	1.2138 × 10^−14^	2.4831 × 10^−15^	0.0000 × 10^0^	1.2138 × 10^−14^	2.4831 × 10^−15^	0.0000 × 10^0^
CESCA	6.7169 × 10^0^	1.9070 × 10^0^	1.0700 × 10^1^	6.7169 × 10^0^	1.9070 × 10^0^	1.0700 × 10^1^
IGWO	4.6777 × 10^−15^	9.0135 × 10^−16^	0.0000 × 10^0^	4.6777 × 10^−15^	9.0135 × 10^−16^	0.0000 × 10^0^
BMWOA	4.6994 × 10^−3^	5.2250 × 10^−3^	1.7612 × 10^−3^	4.6994 × 10^−3^	5.2250 × 10^−3^	1.7612 × 10^−3^
OBLGWO	8.8818 × 10^−16^	0.0000 × 10^0^	0.0000 × 10^0^	8.8818 × 10^−16^	0.0000 × 10^0^	0.0000 × 10^0^
	F13		F14		F15	
	mean	std	mean	mean	std	mean
ISMA	**1.3498 × 10^−32^**	**5.5674 × 10^−48^**	**9.9800 × 10^−1^**	**1.3498 × 10^−32^**	**5.5674 × 10^−48^**	**9.9800 × 10^−1^**
MPEDE	3.2626 × 10^−1^	9.4775 × 10^−1^	9.9800 × 10^−1^	3.2626 × 10^−1^	9.4775 × 10^−1^	9.9800 × 10^−1^
LSHADE	1.1303 × 10^−1^	4.0369 × 10^−1^	9.9800 × 10^−1^	1.1303 × 10^−1^	4.0369 × 10^−1^	9.9800 × 10^−1^
ALCPSO	1.1403 × 10^−2^	3.4415 × 10^−2^	9.9800 × 10^−1^	1.1403 × 10^−2^	3.4415 × 10^−2^	9.9800 × 10^−1^
CLPSO	1.3498 × 10^−32^	5.5674 × 10^−48^	9.9800 × 10^−1^	1.3498 × 10^−32^	5.5674 × 10^−48^	9.9800 × 10^−1^
CESCA	4.2932 × 10^5^	6.0065 × 10^5^	3.0471 × 10^0^	4.2932 × 10^5^	6.0065 × 10^5^	3.0471 × 10^0^
IGWO	1.6832 × 10^−2^	3.2997 × 10^−2^	9.9800 × 10^−1^	1.6832 × 10^−2^	3.2997 × 10^−2^	9.9800 × 10^−1^
BMWOA	1.7335 × 10^−4^	5.7395 × 10^−4^	9.9800 × 10^−1^	1.7335 × 10^−4^	5.7395 × 10^−4^	9.9800 × 10^−1^
OBLGWO	2.4316 × 10^−2^	3.9405 × 10^−2^	9.9800 × 10^−1^	2.4316 × 10^−2^	3.9405 × 10^−2^	9.9800 × 10^−1^
	F16		F17		F18	
	mean	std	mean	mean	std	mean
ISMA	−1.032 × 10^0^	6.9699 × 10^−9^	3.9808 × 10^−1^	−1.032 × 10^0^	6.9699 × 10^−9^	3.9808 × 10^−1^
MPEDE	−1.032 × 10^0^	6.7752 × 10^−16^	3.9789 × 10^−1^	−1.032 × 10^0^	6.7752 × 10^−16^	3.9789 × 10^−1^
LSHADE	−1.032 × 10^0^	6.7752 × 10^−16^	3.9789 × 10^−1^	−1.032 × 10^0^	6.7752 × 10^−16^	3.9789 × 10^−1^
ALCPSO	−1.032 × 10^0^	5.6082 × 10^−16^	3.9789 × 10^−1^	−1.032 × 10^0^	5.6082 × 10^−16^	3.9789 × 10^−1^
CLPSO	−1.032 × 10^0^	6.4539 × 10^−16^	**3.9789 × 10^−1^**	−1.032 × 10^0^	6.4539 × 10^−16^	**3.9789 × 10^−1^**
CESCA	−1.026 × 10^0^	5.9057 × 10^−3^	7.0892 × 10^−1^	−1.026 × 10^0^	5.9057 × 10^−3^	7.0892 × 10^−1^
IGWO	−1.032 × 10^0^	2.2583 × 10^−13^	3.9789 × 10^−1^	−1.032 × 10^0^	2.2583 × 10^−13^	3.9789 × 10^−1^
BMWOA	**−1.031 × 10^0^**	**4.4024 × 10^−16^**	3.9789 × 10^−1^	**−1.031 × 10^0^**	**4.4024 × 10^−16^**	3.9789 × 10^−1^
OBLGWO	−1.032 × 10^0^	9.0832 × 10^−9^	3.9801 × 10^−1^	−1.032 × 10^0^	9.0832 × 10^−9^	3.9801 × 10^−1^
	F19		F20		F21	
	mean	std	mean	mean	std	mean
ISMA	−3.863 × 10^0^	9.7215 × 10^−5^	−3.159 × 10^0^	−3.863 × 10^0^	9.7215 × 10^−5^	−3.159 × 10^0^
MPEDE	−3.863 × 10^0^	2.7101 × 10^−15^	−3.271 × 10^0^	−3.863 × 10^0^	2.7101 × 10^−15^	−3.271 × 10^0^
LSHADE	−3.863 × 10^0^	1.3042 × 10^−4^	−1.952 × 10^0^	−3.863 × 10^0^	1.3042 × 10^−4^	−1.952 × 10^0^
ALCPSO	**−3.862 × 10^0^**	**2.5243 × 10^−15^**	−3.274 × 10^0^	**−3.862 × 10^0^**	**2.5243 × 10^−15^**	−3.274 × 10^0^
CLPSO	−3.863 × 10^0^	2.7101 × 10^−15^	**−3.322 × 10^0^**	−3.863 × 10^0^	2.7101 × 10^−15^	**−3.322 × 10^0^**
CESCA	−3.610 × 10^0^	1.6803 × 10^−1^	−2.176 × 10^0^	−3.610 × 10^0^	1.6803 × 10^−1^	−2.176 × 10^0^
IGWO	−3.863 × 10^0^	1.0500 × 10^−9^	−3.251 × 10^0^	−3.863 × 10^0^	1.0500 × 10^−9^	−3.251 × 10^0^
BMWOA	−3.863 × 10^0^	1.5134 × 10^−14^	−3.290 × 10^0^	−3.863 × 10^0^	1.5134 × 10^−14^	−3.290 × 10^0^
OBLGWO	−3.863 × 10^0^	1.3281 × 10^−6^	−3.223 × 10^0^	−3.863 × 10^0^	1.3281 × 10^−6^	−3.223 × 10^0^
	F22		F23		F24	
	mean	std	mean	mean	std	mean
ISMA	−1.040 × 10^1^	5.9774 × 10^−6^	−1.054 × 10^1^	−1.040 × 10^1^	5.9774 × 10^−6^	−1.054 × 10^1^
MPEDE	−9.542 × 10^0^	2.2747 × 10^0^	−9.817 × 10^0^	−9.542 × 10^0^	2.2747 × 10^0^	−9.817 × 10^0^
LSHADE	−1.023 × 10^1^	9.6292 × 10^−1^	**−1.053 × 10^1^**	−1.023 × 10^1^	9.6292 × 10^−1^	**−1.053 × 10^1^**
ALCPSO	−9.876 × 10^0^	1.6093 × 10^0^	−9.997 × 10^0^	−9.876 × 10^0^	1.6093 × 10^0^	−9.997 × 10^0^
CLPSO	−1.040 × 10^1^	5.7155 × 10^−9^	−1.054 × 10^1^	−1.040 × 10^1^	5.7155 × 10^−9^	−1.054 × 10^1^
CESCA	−1.091 × 10^0^	4.2964 × 10^−1^	−1.172 × 10^0^	−1.091 × 10^0^	4.2964 × 10^−1^	−1.172 × 10^0^
IGWO	−9.166 × 10^0^	2.2815 × 10^0^	−1.018 × 10^1^	−9.166 × 10^0^	2.2815 × 10^0^	−1.018 × 10^1^
BMWOA	**−1.040 × 10^1^**	**9.4634 × 10^−11^**	−1.054 × 10^1^	**−1.040 × 10^1^**	**9.4634 × 10^−11^**	−1.054 × 10^1^
OBLGWO	−1.040 × 10^1^	3.5332 × 10^−5^	−1.054 × 10^1^	−1.040 × 10^1^	3.5332 × 10^−5^	−1.054 × 10^1^
	F25		F26		F27	
	mean	std	mean	mean	std	mean
ISMA	3.4696 × 10^3^	1.5041 × 10^2^	**2.5000 × 10^3^**	3.4696 × 10^3^	1.5041 × 10^2^	**2.5000 × 10^3^**
MPEDE	2.5483 × 10^3^	2.1545 × 10^2^	2.6152 × 10^3^	2.5483 × 10^3^	2.1545 × 10^2^	2.6152 × 10^3^
LSHADE	2.4214 × 10^3^	1.2400 × 10^2^	2.6152 × 10^3^	2.4214 × 10^3^	1.2400 × 10^2^	2.6152 × 10^3^
ALCPSO	2.6317 × 10^3^	1.8339 × 10^2^	2.6153 × 10^3^	2.6317 × 10^3^	1.8339 × 10^2^	2.6153 × 10^3^
CLPSO	**2.4055 × 10^3^**	**8.0140 × 10^1^**	2.6152 × 10^3^	**2.4055 × 10^3^**	**8.0140 × 10^1^**	2.6152 × 10^3^
CESCA	5.5650 × 10^3^	9.4857 × 10^2^	3.0675 × 10^3^	5.5650 × 10^3^	9.4857 × 10^2^	3.0675 × 10^3^
IGWO	2.5661 × 10^3^	1.8331 × 10^2^	2.6206 × 10^3^	2.5661 × 10^3^	1.8331 × 10^2^	2.6206 × 10^3^
BMWOA	2.9003 × 10^3^	1.9433 × 10^2^	2.5005 × 10^3^	2.9003 × 10^3^	1.9433 × 10^2^	2.5005 × 10^3^
OBLGWO	2.6973 × 10^3^	2.3782 × 10^2^	2.6188 × 10^3^	2.6973 × 10^3^	2.3782 × 10^2^	2.6188 × 10^3^
	F28		F29		F30	
	mean	std	mean	mean	std	mean
ISMA	**2.7000 × 10^3^**	**0.0000 × 10^0^**	2.7181 × 10^3^	**2.7000 × 10^3^**	**0.0000 × 10^0^**	2.7181 × 10^3^
MPEDE	2.7112 × 10^3^	4.6410 × 10^0^	2.7202 × 10^3^	2.7112 × 10^3^	4.6410 × 10^0^	2.7202 × 10^3^
LSHADE	2.7056 × 10^3^	3.3938 × 10^0^	2.7104 × 10^3^	2.7056 × 10^3^	3.3938 × 10^0^	2.7104 × 10^3^
ALCPSO	2.7124 × 10^3^	5.0481 × 10^0^	2.7553 × 10^3^	2.7124 × 10^3^	5.0481 × 10^0^	2.7553 × 10^3^
CLPSO	2.7072 × 10^3^	9.5781 × 10^−1^	**2.7004 × 10^3^**	2.7072 × 10^3^	9.5781 × 10^−1^	**2.7004 × 10^3^**
CESCA	2.7206 × 10^3^	8.6833 × 10^0^	2.7123 × 10^3^	2.7206 × 10^3^	8.6833 × 10^0^	2.7123 × 10^3^
IGWO	2.7107 × 10^3^	2.5492 × 10^0^	2.7007 × 10^3^	2.7107 × 10^3^	2.5492 × 10^0^	2.7007 × 10^3^
BMWOA	2.7000 × 10^3^	1.1250 × 10^−2^	2.7006 × 10^3^	2.7000 × 10^3^	1.1250 × 10^−2^	2.7006 × 10^3^
OBLGWO	2.7000 × 10^3^	0.0000 × 10^0^	2.7005 × 10^3^	2.7000 × 10^3^	0.0000 × 10^0^	2.7005 × 10^3^
	F31		F32		F33	
	mean	std	mean	mean	std	mean
ISMA	**3.0000 × 10^3^**	**0.0000 × 10^0^**	**3.1000 × 10^3^**	**3.0000 × 10^3^**	**0.0000 × 10^0^**	**3.1000 × 10^3^**
MPEDE	3.9778 × 10^3^	3.4239 × 10^2^	1.6519 × 10^6^	3.9778 × 10^3^	3.4239 × 10^2^	1.6519 × 10^6^
LSHADE	3.7470 × 10^3^	8.7552 × 10^1^	2.9248 × 10^5^	3.7470 × 10^3^	8.7552 × 10^1^	2.9248 × 10^5^
ALCPSO	4.4793 × 10^3^	5.0276 × 10^2^	2.8922 × 10^6^	4.4793 × 10^3^	5.0276 × 10^2^	2.8922 × 10^6^
CLPSO	3.7271 × 10^3^	8.5165 × 10^1^	3.8465 × 10^3^	3.7271 × 10^3^	8.5165 × 10^1^	3.8465 × 10^3^
CESCA	5.4621 × 10^3^	2.9312 × 10^2^	1.6432 × 10^7^	5.4621 × 10^3^	2.9312 × 10^2^	1.6432 × 10^7^
IGWO	3.7942 × 10^3^	1.0332 × 10^2^	8.4824 × 10^5^	3.7942 × 10^3^	1.0332 × 10^2^	8.4824 × 10^5^
BMWOA	3.0001 × 10^3^	1.8250 × 10^−1^	3.8977 × 10^5^	3.0001 × 10^3^	1.8250 × 10^−1^	3.8977 × 10^5^
OBLGWO	3.5344 × 10^3^	4.8730 × 10^2^	3.4895 × 10^6^	3.5344 × 10^3^	4.8730 × 10^2^	3.4895 × 10^6^

**Table A9:** Wilcoxon signed-rank test results between the ISMA and other advanced algorithms.

Function	MPEDE	LSHADE	ALCPSO	CLPSO	CESCA	IGWO	BMWOA	OBLGWO
F1	1.7344 × 10^−6^	1.7333 × 10^−6^	1.7333 × 10^−6^	1.7344 × 10^−6^	1.7344 × 10^−6^	1.0000 × 10^0^	1.7344 × 10^−6^	1.0000 × 10^0^
F2	1.7344 × 10^−6^	1.7344 × 10^−6^	1.7344 × 10^−6^	1.7344 × 10^−6^	1.7344 × 10^−6^	1.7344 × 10^−6^	1.7344 × 10^−6^	1.7344 × 10^−6^
F3	1.7344 × 10^−6^	1.7344 × 10^−6^	1.7344 × 10^−6^	1.7344 × 10^−6^	1.7344 × 10^−6^	1.7344 × 10^−6^	1.7344 × 10^−6^	2.5000 × 10^−1^
F4	1.7344 × 10^−6^	1.7344 × 10^−6^	1.7344 × 10^−6^	1.7344 × 10^−6^	1.7344 × 10^−6^	1.7344 × 10^−6^	1.7344 × 10^−6^	3.7896 × 10^−6^
F5	8.1806 × 10^−5^	5.9829 × 10^−2^	1.7344 × 10^−6^	1.7344 × 10^−6^	1.7344 × 10^−6^	1.7344 × 10^−6^	1.7344 × 10^−6^	1.7344 × 10^−6^
F6	3.5657 × 10^−4^	2.4414 × 10^−4^	1.7333 × 10^−6^	1.0000 × 10^0^	1.7344 × 10^−6^	1.7344 × 10^−6^	1.7344 × 10^−6^	1.7344 × 10^−6^
F7	1.7344 × 10^−6^	1.7344 × 10^−6^	1.7344 × 10^−6^	1.7344 × 10^−6^	1.7344 × 10^−6^	5.7924 × 10^−5^	1.7344 × 10^−6^	3.1123 × 10^−5^
F8	1.4831 × 10^−3^	1.4591 × 10^−3^	1.4835 × 10^−3^	1.3642 × 10^−3^	1.4557 × 10^−3^	1.4839 × 10^−3^	1.4839 × 10^−3^	1.4839 × 10^−3^
F9	1.7300 × 10^−6^	5.0136 × 10^−6^	1.7344 × 10^−6^	1.0000 × 10^0^	1.7344 × 10^−6^	1.0000 × 10^0^	1.7344 × 10^−6^	1.0000 × 10^0^
F10	1.7203 × 10^−6^	8.7824 × 10^−7^	1.7041 × 10^−6^	1.0651 × 10^−6^	1.7344 × 10^−6^	1.0135 × 10^−7^	1.7344 × 10^−6^	1.0000 × 10^0^
F11	1.9472 × 10^−4^	3.9586 × 10^−5^	1.3163 × 10^−4^	1.0000 × 10^0^	1.7333 × 10^−6^	1.0000 × 10^0^	1.7333 × 10^−6^	1.0000 × 10^0^
F12	2.6499 × 10^−5^	1.7948 × 10^−5^	1.7311 × 10^−6^	1.0000 × 10^0^	1.7344 × 10^−6^	1.7344 × 10^−6^	1.7344 × 10^−6^	1.7344 × 10^−6^
F13	5.2772 × 10^−5^	4.0204 × 10^−4^	1.7062 × 10^−6^	1.0000 × 10^0^	1.7344 × 10^−6^	1.7344 × 10^−6^	1.7344 × 10^−6^	1.7344 × 10^−6^
F14	1.0000 × 10^0^	1.0000 × 10^0^	1.0000 × 10^0^	1.0000 × 10^0^	1.7344 × 10^−6^	4.1722 × 10^−7^	3.9063 × 10^−3^	1.7344 × 10^−6^
F15	1.4795 × 10^−2^	1.9209 × 10^−6^	2.7653 × 10^−3^	1.7344 × 10^−6^	1.7344 × 10^−6^	5.9836 × 10^−2^	2.7653 × 10^−3^	1.8519 × 10^−2^
F16	1.7344 × 10^−6^	1.7344 × 10^−6^	1.7344 × 10^−6^	1.7344 × 10^−6^	1.7344 × 10^−6^	1.7344 × 10^−6^	1.7344 × 10^−6^	1.1748 × 10^−2^
F17	1.7344 × 10^−6^	1.7344 × 10^−6^	1.7344 × 10^−6^	1.7344 × 10^−6^	1.7344 × 10^−6^	1.7344 × 10^−6^	1.7344 × 10^−6^	2.1827 × 10^−2^
F18	1.7344 × 10^−6^	1.7344 × 10^−6^	1.7344 × 10^−6^	1.7344 × 10^−6^	1.3059 × 10^−1^	1.7344 × 10^−6^	1.7344 × 10^−6^	1.7344 × 10^−6^
F19	1.7344 × 10^−6^	3.1123 × 10^−5^	1.7344 × 10^−6^	1.7344 × 10^−6^	1.7344 × 10^−6^	1.7344 × 10^−6^	1.7344 × 10^−6^	2.3534 × 10^−6^
F20	3.8822 × 10^−6^	1.9152 × 10^−1^	3.8822 × 10^−6^	1.7344 × 10^−6^	1.9209 × 10^−6^	8.4661 × 10^−6^	6.3391 × 10^−6^	2.2248 × 10^−4^
F21	6.4352 × 10^−1^	1.6503 × 10^−1^	1.4795 × 10^−2^	7.7309 × 10^−3^	1.7344 × 10^−6^	1.7344 × 10^−6^	1.7344 × 10^−6^	1.7344 × 10^−6^
F22	1.4795 × 10^−2^	3.1123 × 10^−5^	2.7653 × 10^−3^	1.7344 × 10^−6^	1.7344 × 10^−6^	4.9498 × 10^−2^	1.7344 × 10^−6^	1.7344 × 10^−6^
F23	2.7653 × 10^−3^	1.7344 × 10^−6^	2.7653 × 10^−3^	1.7344 × 10^−6^	1.7344 × 10^−6^	6.8836 × 10^−1^	1.7344 × 10^−6^	2.6033 × 10^−6^
F24	1.7344 × 10^−6^	1.7344 × 10^−6^	1.7344 × 10^−6^	1.7344 × 10^−6^	2.6033 × 10^−6^	1.7344 × 10^−6^	1.7344 × 10^−6^	1.7344 × 10^−6^
F25	1.7344 × 10^−6^	1.7344 × 10^−6^	1.7344 × 10^−6^	1.7344 × 10^−6^	1.7344 × 10^−6^	1.7344 × 10^−6^	1.9209 × 10^−6^	1.7344 × 10^−6^
F26	4.3205 × 10^−8^	6.7988 × 10^−8^	1.7344 × 10^−6^	1.7333 × 10^−6^	1.7333 × 10^−6^	1.7333 × 10^−6^	1.7333 × 10^−6^	1.7344 × 10^−6^
F27	1.7344 × 10^−6^	1.7344 × 10^−6^	1.7344 × 10^−6^	1.7344 × 10^−6^	1.7344 × 10^−6^	1.7344 × 10^−6^	1.7344 × 10^−6^	1.0000 × 10^0^
F28	1.7344 × 10^−6^	1.7344 × 10^−6^	1.7344 × 10^−6^	1.7344 × 10^−6^	1.7344 × 10^−6^	1.7344 × 10^−6^	1.7344 × 10^−6^	1.0000 × 10^0^
F29	7.8647 × 10^−2^	1.4839 × 10^−3^	1.4139 × 10^−1^	1.7344 × 10^−6^	2.5637 × 10^−2^	1.7344 × 10^−6^	1.7344 × 10^−6^	1.7344 × 10^−6^
F30	1.7344 × 10^−6^	1.7344 × 10^−6^	1.7344 × 10^−6^	1.7344 × 10^−6^	1.7344 × 10^−6^	1.7344 × 10^−6^	1.7344 × 10^−6^	2.5000 × 10^−1^
F31	1.7344 × 10^−6^	1.7344 × 10^−6^	1.7344 × 10^−6^	1.7344 × 10^−6^	1.7344 × 10^−6^	1.7344 × 10^−6^	1.7344 × 10^−6^	2.9305 × 10^−4^
F32	1.7344 × 10^−6^	1.7344 × 10^−6^	1.7344 × 10^−6^	1.7344 × 10^−6^	1.7344 × 10^−6^	1.7344 × 10^−6^	1.7344 × 10^−6^	1.7344 × 10^−6^
F33	1.7344 × 10^−6^	1.7344 × 10^−6^	1.7344 × 10^−6^	1.7344 × 10^−6^	1.7344 × 10^−6^	1.7344 × 10^−6^	1.7344 × 10^−6^	1.7344 × 10^−6^
+/=/−	22/3/8	20/4/9	23/2/8	16/6/11	30/1/2	19/5/9	21/0/12	16/8/9

**Table A10:** Average ranking values using the Friedman test.

Algorithm	ISMA	MPEDE	LSHADE	ALCPSO	CLPSO	CESCA	IGWO	BMWOA	OBLGWO
AVR	**3.7075758**	4.0257576	4.1979798	5.1949495	3.8792929	8.8474747	5.0984848	5.080303	4.9681818
rank	**1**	3	4	8	2	9	7	6	5

**Table A11:** The descriptions of two types of TFs.

S-Shaped Family		
Name	TFs	Graphs
TFS1	$T\left(x_{i}^{j}\left(t\right)\right)=\frac{1}{1+exp^{-2x_{i}^{j}\left(t\right)}}$	
TFS2	$T\left(x_{i}^{j}\left(t\right)\right)=\frac{1}{1+exp^{-2x_{i}^{j}\left(t\right)}}$	
TFS3	$T\left(x_{i}^{j}\left(t\right)\right)=\frac{1}{1+exp^{-2x_{i}^{j}\left(t\right)}}$	
TFS4	$T\left(x_{i}^{j}\left(t\right)\right)=\frac{1}{1+exp^{-2x_{i}^{j}\left(t\right)}}$	
**V-Shaped Family**		
**Name**	**TFs**	*Graphs*
TFV1	$T\left(x_{i}^{j}\left(t\right)\right)=\left|erf\left(\frac{\sqrt{\pi }}{2}x_{i}^{j}\left(t\right)\right)\right|$ $=\left|\frac{\sqrt{\pi }}{2}\int_{0}^{\frac{(\sqrt{\pi }}{2)x_{i\cdot }^{j}}}\left(t\right)e^{-t^{2}}dt\right|$	
TFV2	$T\left(x_{i}^{j}\left(t\right)\right)=\left|tanh\;tanh\;x_{i}^{j}\left(t\right)\;\right|$	
TFV3	$T\left(x_{i}^{j}\left(t\right)\right)=\left|\frac{x_{i}^{j}\left(t\right)}{\sqrt{1+x_{i}^{j}{\left(t\right)}^{2}}}\right|$	
TFV4	$T\left(x_{i}^{j}\left(t\right)\right)=\left|\frac{2}{\pi }arctan\left(\frac{\sqrt{\pi }}{2}x_{i}^{j}\left(t\right)\right)\right|$	

Note: $x_{i}^{j}\left(t\right)$ denotes the i-th element on j-th dimension in the position vector.

**Table A12:** Characteristics of gene expression datasets.

Datasets	Samples	Genes	Categories
**Colon**	62	2000	2
**SRBCT**	83	2309	4
**Leukemia**	72	7131	2
**Brain_Tumor1**	90	5920	5
**Brain_Tumor2**	50	10,367	4
**CNS**	60	7130	2
**DLBCL**	77	5470	4
**Leukemia1**	72	5328	5
**Leukemia2**	72	11,225	3
**Lung_Cancer**	203	12,601	3
**Prostate_Tumor**	102	10,509	2
**Tumors_9**	60	5726	9
**Tumors_11**	174	12,533	11
**Tumors_14**	308	15,009	26

**Table A13:** Overall results of eight versions of BISMA according to S-shaped and V-shaped TFs in terms of average number of the selected genes.

Datasets	Metrics	BISMA_S1	BISMA_S2	BISMA_S3	BISMA_S4	BISMA_V1	BISMA_V2	BISMA_V3	BISMA_V4
**Colon**	std	143.6448	157.4435	173.4187	162.0243	**0.4216**	0.9718	0.6992	0.6992
	avg	307.5000	464.5000	476.5000	498.0000	**1.0000**	1.0000	1.0000	1.0000
**SRBCT**	std	138.2114	95.9528	156.2727	154.4375	2.9515	2.9364	1.9322	**1.4337**
	avg	376.5000	465.5000	566.0000	565.0000	4.0000	5.0000	4.5000	**4.5000**
**Leukemia**	std	589.3556	296.6164	135.8554	64.6241	0.9487	1.2517	0.3162	**0.3162**
	avg	1595.5000	1359.0000	1738.5000	1755.0000	1.0000	1.0000	1.0000	**1.0000**
**Brain_Tumor1**	std	926.7275	778.2962	44.3653	560.9920	147.6392	8.0939	**11.1679**	19.3724
	avg	1050.0000	1319.5000	1451.5000	1461.5000	2.0000	3.0000	**2.5000**	2.5000
**Brain_Tumor2**	std	755.7944	978.0951	955.6762	430.0868	1.7512	0.9944	1.2293	**0.4831**
	avg	1938.0000	2509.5000	2510.0000	2529.5000	1.0000	2.0000	1.5000	**1.0000**
**CNS**	std	504.4472	867.2775	732.4766	489.2598	2.2136	0.5164	**0.0000**	0.4216
	avg	1685.0000	1720.5000	1805.0000	1935.0000	1.0000	1.0000	**1.0000**	1.0000
**DLBCL**	std	292.2214	169.4024	129.8839	79.6573	**0.0000**	0.6750	0.3162	0.6325
	avg	490.5000	1295.0000	1334.5000	1371.5000	**1.0000**	1.0000	1.0000	1.0000
**Leukemia1**	std	348.2874	536.8715	66.7750	77.7810	1.3499	1.8135	**1.1005**	1.2472
	avg	1163.0000	1271.5000	1283.0000	1328.5000	2.0000	2.0000	**2.0000**	2.0000
**Leukemia2**	std	731.5217	497.7822	232.6141	929.4172	3.7357	1.6633	**1.4142**	1.4181
	avg	1255.5000	2532.5000	2673.5000	2737.5000	3.0000	2.5000	**1.5000**	3.0000
**Lung_Cancer**	std	1191.4138	1241.8645	1162.5447	623.9975	19.8161	**16.1593**	29.0746	93.8666
	avg	3066.0000	3122.0000	3111.0000	3162.0000	23.5000	**19.0000**	16.5000	15.5000
**Prostate_Tumor**	std	1573.8463	1270.5976	1119.6290	1279.6201	6.2405	37.9867	**1.0750**	1.8529
	avg	2540.0000	2709.0000	2631.5000	2760.5000	3.5000	2.0000	**2.5000**	2.5000
**Tumors_9**	std	785.7851	856.2383	533.6090	595.3492	243.1681	**42.0502**	595.2484	139.8144
	avg	1376.5000	1409.5000	1698.0000	1421.0000	1.0000	**2.0000**	2.5000	4.0000
**Tumors_11**	std	1040.6752	1660.6726	1391.3213	1285.5454	**108.9483**	288.1741	948.9861	248.4647
	avg	3118.5000	4607.0000	4642.0000	3287.0000	**210.0000**	304.5000	374.5000	233.0000
**Tumors_14**	std	2353.3411	1657.2601	974.4708	1551.2076	1520.8509	930.6287	**618.4779**	966.3795
	avg	4920.0000	7469.0000	7450.0000	6775.0000	1143.5000	760.5000	**540.5000**	569.5000
ARV		5.7143	6.3893	6.8143	7.0393	2.5286	2.6536	2.4464	**2.4143**
Rank		5	6	7	8	3	4	2	**1**

**Table A14:** Overall results of eight versions of BISMA according to S-shaped and V-shaped TFs in terms of average number of average error rate.

Datasets	Metrics	BISMA_S1	BISMA_S2	BISMA_S3	BISMA_S4	BISMA_V1	BISMA_V2	BISMA_V3	BISMA_V4
**Colon**	std	1.305 × 10^−1^	1.399 × 10^−1^	1.620 × 10^−1^	1.042 × 10^−1^	**0.000 × 10^0^**	**0.000 × 10^0^**	**0.000 × 10^0^**	**0.000 × 10^0^**
	avg	0.1429	0.1667	0.1667	0.1548	**0.0000**	**0.0000**	**0.0000**	**0.0000**
**SRBCT**	std	0.000 × 10^0^	0.000 × 10^0^	0.000 × 10^0^	0.000 × 10^0^	**0.000 × 10^0^**	**0.000 × 10^0^**	**0.000 × 10^0^**	**0.000 × 10^0^**
	avg	0.0000	0.0000	0.0000	0.0000	**0.0000**	**0.0000**	**0.0000**	**0.0000**
**Leukemia**	std	0.000 × 10^0^	0.000 × 10^0^	0.000 × 10^0^	0.000 × 10^0^	**0.000 × 10^0^**	**0.000 × 10^0^**	**0.000 × 10^0^**	**0.000 × 10^0^**
	avg	0.0000	0.0000	0.0000	0.0000	**0.0000**	**0.0000**	**0.0000**	**0.0000**
**Brain_Tumor1**	std	5.463 × 10^−2^	5.604 × 10^−2^	7.147 × 10^−2^	5.520 × 10^−2^	3.162 × 10^−2^	3.162 × 10^−2^	3.162 × 10^−2^	**0.000 × 10^0^**
	avg	0.0000	0.0000	0.0500	0.0500	0.0000	0.0000	0.0000	**0.0000**
**Brain_Tumor2**	std	9.088 × 10^−2^	8.051 × 10^−2^	1.370 × 10^−1^	8.051 × 10^−2^	**0.000 × 10^0^**	**0.000 × 10^0^**	**0.000 × 10^0^**	**0.000 × 10^0^**
	avg	0.0000	0.0000	0.0000	0.0000	**0.0000**	**0.0000**	**0.0000**	**0.0000**
**CNS**	std	8.794 × 10^−2^	1.466 × 10^−1^	8.607 × 10^−2^	1.528 × 10^−1^	**0.000 × 10^0^**	**0.000 × 10^0^**	**0.000 × 10^0^**	**0.000 × 10^0^**
	avg	0.1548	0.0000	0.0000	0.1548	**0.0000**	**0.0000**	**0.0000**	**0.0000**
**DLBCL**	std	3.953 × 10^−2^	4.518 × 10^−2^	0.000 × 10^0^	0.000 × 10^0^	**0.000 × 10^0^**	**0.000 × 10^0^**	**0.000 × 10^0^**	**0.000 × 10^0^**
	avg	0.0000	0.0000	0.0000	0.0000	**0.0000**	**0.0000**	**0.0000**	**0.0000**
**Leukemia1**	std	0.000 × 10^0^	0.000 × 10^0^	0.000 × 10^0^	0.000 × 10^0^	**0.000 × 10^0^**	**0.000 × 10^0^**	**0.000 × 10^0^**	**0.000 × 10^0^**
	avg	0.0000	0.0000	0.0000	0.0000	**0.0000**	**0.0000**	**0.0000**	**0.0000**
**Leukemia2**	std	0.000 × 10^0^	4.518 × 10^−2^	4.518 × 10^−2^	0.000 × 10^0^	**0.000 × 10^0^**	**0.000 × 10^0^**	**0.000 × 10^0^**	**0.000 × 10^0^**
	avg	0.0000	0.0000	0.0000	0.0000	**0.0000**	**0.0000**	**0.0000**	**0.0000**
**Lung_Cancer**	std	2.528 × 10^−2^	2.561 × 10^−2^	2.491 × 10^−2^	3.310 × 10^−2^	0.000 × 10^0^	1.506 × 10^−2^	0.000 × 10^0^	**0.000 × 10^0^**
	avg	0.0000	0.0238	0.0000	0.0000	0.0000	0.0000	0.0000	**0.0000**
**Prostate_Tumor**	std	6.449 × 10^−2^	5.020 × 10^−2^	7.071 × 10^−2^	5.182 × 10^−2^	3.162 × 10^−2^	0.000 × 10^0^	0.000 × 10^0^	**0.000 × 10^0^**
	avg	0.0000	0.0909	0.0000	0.0455	0.0000	0.0000	0.0000	**0.0000**
**Tumors_9**	std	7.313 × 10^−2^	1.315 × 10^−1^	6.325 × 10^−2^	1.406 × 10^−1^	5.271 × 10^−2^	0.000 × 10^0^	0.000 × 10^0^	**0.000 × 10^0^**
	avg	0.0000	0.0000	0.0000	0.0000	0.0000	0.0000	0.0000	**0.0000**
**Tumors_11**	std	4.353 × 10^−2^	4.395 × 10^−2^	5.206 × 10^−2^	4.678 × 10^−2^	2.886 × 10^−2^	2.413 × 10^−2^	2.975 × 10^−2^	1.757 × 10^−2^
	avg	0.0556	0.0590	0.0572	0.0572	0.0000	0.0000	0.0000	0.0000
**Tumors_14**	std	4.856 × 10^−2^	1.028 × 10^−1^	5.861 × 10^−2^	4.875 × 10^−2^	4.411 × 10^−2^	7.900 × 10^−2^	3.750 × 10^−2^	6.582 × 10^−2^
	avg	0.2952	0.2540	0.2971	0.2833	0.2500	0.2374	0.2457	0.2379
ARV		5.1107	5.1107	5.0429	5.0571	4.9857	4.0429	3.9571	3.9429
Rank		8	8	6	7	5	4	3	2

**Table A15:** Overall results of eight versions of BISMA according to S-shaped and V-shaped TFs in terms of average number of average fitness.

Datasets	Metrics	BISMA_S1	BISMA_S2	BISMA_S3	BISMA_S4	BISMA_V1	BISMA_V2	BISMA_V3	BISMA_V4
**Colon**	std	1.2251 × 10^−1^	1.3115 × 10^−1^	1.5248 × 10^−1^	9.9228 × 10^−2^	**1.0500 × 10^−5^**	2.4300 × 10^−5^	1.7500 × 10^−5^	1.7500 × 10^−5^
	avg	0.14415	0.16695	0.16966	0.16554	**2.50 × 10^−5^**	2.50 × 10^−5^	2.50 × 10^−5^	2.50 × 10^−5^
**SRBCT**	std	2.9942 × 10^−3^	2.0787 × 10^−3^	3.3855 × 10^−3^	3.3457 × 10^−3^	6.3900 × 10^−5^	6.3600 × 10^−5^	4.1900 × 10^−5^	**3.1100 × 10^−5^**
	avg	0.0081564	0.010084	0.012262	0.01224	8.67 × 10^−5^	0.00010832	9.75 × 10^−5^	**9.75 × 10^−5^**
**Leukemia**	std	4.1329 × 10^−3^	2.0801 × 10^−3^	9.5270 × 10^−4^	4.5318 × 10^−4^	6.6500 × 10^−6^	8.7800 × 10^−6^	2.2200 × 10^−6^	**2.2200 × 10^−6^**
	avg	0.011189	0.0095302	0.012191	0.012307	7.01 × 10^−6^	7.01 × 10^−6^	7.01 × 10^−6^	**7.01 × 10^−6^**
**Brain_Tumor1**	std	5.1602 × 10^−2^	5.4124 × 10^−2^	6.7843 × 10^−2^	5.1574 × 10^−2^	2.9920 × 10^−2^	3.0033 × 10^−2^	3.0031 × 10^−2^	**1.6362 × 10^−4^**
	avg	0.018758	0.018163	0.059215	0.06541	1.69 × 10^−5^	2.53 × 10^−5^	2.11 × 10^−5^	**2.11 × 10^−5^**
**Brain_Tumor2**	std	8.4413 × 10^−2^	7.4520 × 10^−2^	1.3015 × 10^−1^	7.6131 × 10^−2^	8.4500 × 10^−6^	4.8000 × 10^−6^	5.9300 × 10^−6^	**2.3300 × 10^−6^**
	avg	0.012262	0.015231	0.012441	0.013635	4.82 × 10^−6^	9.65 × 10^−6^	7.23 × 10^−6^	**4.82 × 10^−6^**
**CNS**	std	8.4987 × 10^−2^	1.3856 × 10^−1^	8.0292 × 10^−2^	1.4740 × 10^−1^	1.5500 × 10^−5^	3.6200 × 10^−6^	**0.0000 × 10^0^**	2.9600 × 10^−6^
	avg	0.1548	0.018712	0.023061	0.1594	7.01 × 10^−6^	7.01 × 10^−6^	**7.01 × 10^−6^**	7.01 × 10^−6^
**DLBCL**	std	3.7662 × 10^−2^	4.3039 × 10^−2^	1.1875 × 10^−3^	7.2826 × 10^−4^	**0.0000 × 10^0^**	6.1700 × 10^−6^	2.8900 × 10^−6^	5.7800 × 10^−6^
	avg	0.0044844	0.012009	0.012201	0.012539	**9.14 × 10^−6^**	9.14 × 10^−6^	9.14 × 10^−6^	9.14 × 10^−6^
**Leukemia1**	std	3.2691 × 10^−3^	5.0392 × 10^−3^	6.2676 × 10^−4^	7.3006 × 10^−4^	1.2700 × 10^−5^	1.7000 × 10^−5^	**1.0300 × 10^−5^**	1.1700 × 10^−5^
	avg	0.010916	0.011934	0.012042	0.012469	1.88 × 10^−5^	1.88 × 10^−5^	**1.88 × 10^−5^**	1.88 × 10^−5^
**Leukemia2**	std	3.2584 × 10^−3^	4.3716 × 10^−2^	4.3135 × 10^−2^	4.1399 × 10^−3^	1.6600 × 10^−5^	7.4100 × 10^−6^	**6.3000 × 10^−6^**	6.3200 × 10^−6^
	avg	0.0055924	0.011281	0.011909	0.012194	1.34 × 10^−5^	1.11 × 10^−5^	**6.68 × 10^−6^**	1.34 × 10^−5^
**Lung_Cancer**	std	2.3808 × 10^−2^	2.2944 × 10^−2^	2.2084 × 10^−2^	3.1035 × 10^−2^	7.8600 × 10^−5^	1.4293 × 10^−2^	**1.1538 × 10^−4^**	3.7249 × 10^−4^
	avg	0.018605	0.04004	0.022837	0.013115	9.33 × 10^−5^	8.73 × 10^−5^	**6.55 × 10^−5^**	6.15 × 10^−5^
**Prostate_Tumor**	std	6.2632 × 10^−2^	4.4868 × 10^−2^	6.4827 × 10^−2^	4.9589 × 10^−2^	3.0037 × 10^−2^	1.8073 × 10^−4^	**5.1100 × 10^−6^**	8.8200 × 10^−6^
	avg	0.018427	0.098843	0.024919	0.06217	2.85 × 10^−5^	9.52 × 10^−6^	**1.19 × 10^−5^**	1.19 × 10^−5^
**Tumors_9**	std	7.3201 × 10^−2^	1.2925 × 10^−1^	6.0930 × 10^−2^	1.3705 × 10^−1^	5.1706 × 10^−2^	**3.6719 × 10^−4^**	5.1978 × 10^−3^	1.2209 × 10^−3^
	avg	0.012256	0.012308	0.014827	0.012408	8.73 × 10^−6^	**1.75 × 10^−5^**	2.18 × 10^−5^	3.49 × 10^−5^
**Tumors_11**	std	4.0291 × 10^−2^	4.4019 × 10^−2^	4.8341 × 10^−2^	4.3815 × 10^−2^	2.7431 × 10^−2^	2.2469 × 10^−2^	2.7845 × 10^−2^	**1.7275 × 10^−2^**
	avg	0.0646	0.074911	0.071693	0.06889	0.0013903	0.0019768	0.0046557	**0.00092955**
**Tumors_14**	std	4.2144 × 10^−2^	9.9535 × 10^−2^	5.5160 × 10^−2^	4.2749 × 10^−2^	4.2056 × 10^−2^	7.3598 × 10^−2^	**3.5842 × 10^−2^**	6.0899 × 10^−2^
	avg	0.30311	0.26614	0.30706	0.28783	0.24017	0.22696	**0.23548**	0.22735
ARV		5.9786	5.9786	6.2214	6.6214	6.6929	2.7036	2.7536	2.6036
Rank		5	5	6	7	8	3	4	2

**Table A16:** Overall results of eight versions of BISMA according to S-shaped and V-shaped TFs in terms of average number of average computational time.

Datasets	Metrics	BISMA_S1	BISMA_S2	BISMA_S3	BISMA_S4	BISMA_V1	BISMA_V2	BISMA_V3	BISMA_V4
**Colon**	std	1.2191	1.3052	2.4355	1.4757	1.1938	1.4909	**1.2686**	1.3287
	avg	85.9626	90.0505	121.3363	89.5739	94.0297	84.1583	**82.0512**	82.549
**SRBCT**	std	1.5885	1.8773	2.8525	1.084	1.8508	2.45	**3.0216**	2.414
	avg	102.6595	105.9233	153.5824	106.7198	110.4927	101.3722	**94.9202**	98.0153
**Leukemia**	std	4.6463	7.1863	8.4485	4.176	5.6477	8.9345	7.1624	**5.6551**
	avg	288.1026	369.0878	418.859	300.3361	312.7438	281.3737	263.277	**262.0363**
**Brain_Tumor1**	std	15.0486	5.518	5.9123	3.6483	6.5985	7.0206	5.2887	**9.7095**
	avg	257.1141	329.2853	355.1649	265.6545	268.0102	235.1687	226.2937	**221.4106**
**Brain_Tumor2**	std	26.0923	16.4663	5.829	5.9109	5.7821	6.9892	8.4514	**4.4363**
	avg	394.7483	557.1407	417.7936	408.0532	429.047	378.0446	403.66	**366.1612**
**CNS**	std	18.5258	8.2571	4.9416	4.7855	5.2549	5.1788	6.3485	**2.5764**
	avg	282.115	399.2233	297.2468	292.9844	305.7291	270.9286	305.3575	**257.4227**
**DLBCL**	std	13.4459	7.3986	3.7698	3.2965	6.6934	6.881	**5.9037**	6.3564
	avg	229.0604	318.173	239.9178	235.4501	243.1863	222.2096	**206.4178**	207.6545
**Leukemia1**	std	13.0915	7.1145	4.2194	3.5637	4.3786	5.3226	3.4246	**4.1366**
	avg	221.6625	306.661	230.06	226.9516	236.7801	206.948	201.3261	**199.8014**
**Leukemia2**	std	27.9557	27.8952	7.3565	6.2185	9.6514	10.0649	7.2691	**9.5984**
	avg	454.5811	626.5679	467.8857	467.3684	482.2521	424.8834	411.641	**408.7297**
**Lung_Cancer**	std	40.0181	14.4133	21.3431	26.7837	47.3963	37.8825	48.654	**32.9511**
	avg	835.7816	1064.939	847.6348	828.0133	677.3208	558.4493	534.8904	**521.5364**
**Prostate_Tumor**	std	25.1417	10.4573	6.7311	10.3808	19.9367	16.2087	12.0796	**24.8174**
	avg	470.1901	659.3352	485.7299	477.1534	464.5947	415.6169	390.0605	**389.1298**
**Tumors_9**	std	13.5588	8.8614	3.2316	4.0011	2.6109	4.0259	4.3268	**3.487**
	avg	231.0626	333.3597	240.6433	238.7621	246.7118	220.015	208.5856	**206.8161**
**Tumors_11**	std	39.8624	15.6572	18.8506	15.9373	46.4145	36.5902	20.4801	**15.6274**
	avg	744.1785	985.7713	758.463	752.3035	630.7326	555.8758	502.5768	**483.1388**
**Tumors_14**	std	73.1984	62.0491	69.1097	103.7124	77.7274	74.0669	49.1133	**57.7599**
	avg	1560.365	1901.44	1556.638	1541.604	1087.476	880.2872	760.1826	**723.8812**
ARV		4.7	7.5143	6.4071	5.0571	5.8929	2.8643	2.1357	**1.4286**
Rank		4	8	7	5	6	3	2	**1**

**Table A17:** Parameter settings.

Optimizers	Parameters	Value
bGWO	$a_{max}$	2
	$a_{min}$	0
BPSO	Min inertia weight	0.4
	Min inertia weight	0.9
	c1, c2	0.2
bWOA	$a_{max}$	2
	$a_{min}$	0

**Table A18:** Comparison of BISMA with other gene selection optimizers in terms of average number of the selected genes.

Datasets	Metrics	BISMA	BSMA	bGWO	BGSA	BPSO	bALO	BBA	BSSA	bWOA
**Colon**	std	**0.5164**	29.5727	15.9753	23.2178	18.7901	26.9081	57.2076	413.9399	1.6499
	avg	**1**	46	153.5	769	899	876	818	424.5	2
**SRBCT**	std	**1.2649**	20.5721	15.2567	28.0515	17.2321	21.7348	88.7612	234.9426	1.8974
	avg	**3**	33.5	192	898.5	1023	996	936	1073.5	4
**Leukemia**	std	**0.42164**	21.9699	41.075	22.2264	31.8531	27.3595	180.2885	1254.8997	0.91894
	avg	**1**	36	791.5	3106	3354	3288	2850	3427	2
**Brain_Tumor1**	std	3.1429	78.3272	37.8001	45.6636	31.3739	42.0132	104.9288	1333.051	**1.2649**
	avg	3.5	65	631	2559	2766	2737	2449.5	2646.5	**3**
**Brain_Tumor2**	std	1.7029	240.6062	75.5373	55.0019	55.9691	46.9871	135.9838	2454.5883	**1.1785**
	avg	2.5	156	1148.5	4672.5	4914.5	4864.5	4209	2946.5	**2.5**
**CNS**	std	**0.31623**	136.7067	42.7265	96.6304	35.9623	50.9117	198.0223	1551.1952	3.2335
	avg	**1**	87.5	852	3171	3386.5	3344.5	2985	3293	2
**DLBCL**	std	**0.42164**	33.4865	23.7957	48.9182	24.6162	37.7601	156.4013	833.0272	0.99443
	avg	**1**	40.5	571.5	2329.5	2522.5	2489	2245	2625.5	2
**Leukemia1**	std	**0.8165**	25.3588	33.1832	39.1324	20.8017	31.6665	190.6413	1124.43	1.2649
	avg	**2**	40	550.5	2303	2473.5	2419	2132	2538.5	3.5
**Leukemia2**	std	**1.2649**	22.3617	46.4113	57.6102	51.1196	42.9973	252.5475	2534.8708	1.1972
	avg	**2.5**	55	1245.5	5021.5	5320.5	5272.5	4592	5412.5	3
**Lung_Cancer**	std	27.247	240.5198	66.0041	77.9308	42.3663	48.9689	688.2611	2587.9333	**13.898**
	avg	10	172	1504	5750.5	6030	5947.5	5097.5	6092	**5.5**
**Prostate_Tumor**	std	**1.4181**	234.6364	63.2583	109.3395	83.1836	39.8112	191.4855	2202.9629	1.792
	avg	**2**	181.5	1262.5	4772.5	5029	4955.5	4401.5	5041	3
**Tumors_9**	std	102.7665	812.1526	43.0834	71.0286	45.3878	37.3722	171.1166	1120.278	**3.0258**
	avg	8	174	674	2529	2732.5	2655.5	2376.5	2750	**3**
**Tumors_11**	std	231.5253	558.048	45.2396	142.4798	102.5773	88.8522	190.7012	1889.8508	**113.9361**
	avg	235.5	497	1596.5	5776.5	6080.5	5968.5	5281.5	6134.5	**110.5**
**Tumors_14**	std	681.716	2562.6438	127.5985	132.4234	80.2649	77.5818	187.6832	61.4366	**664.218**
	avg	682	1469	2382.5	7337.5	7401	7357.5	6349.5	7426.5	**565**
ARV		**1.4643**	3.1357	4.1714	6.2643	8.175	7.5107	5.5286	7.1286	1.6214
Rank		**1**	3	4	6	9	8	5	7	2

**Table A19:** Comparison of BISMA with other gene selection optimizers in terms of average error rate.

Datasets	Metrics	BISMA	BSMA	bGWO	BGSA	BPSO	bALO	BBA	BSSA	bWOA
**Colon**	std	**0.0000**	0.0527	0.1162	0.1925	0.1229	0.2222	0.1592	0.1554	0.0000
	avg	**0.0000**	0.0000	0.0000	0.0833	0.1667	0.0833	0.2262	0.0714	0.0000
**SRBCT**	std	**0.0000**	0.0000	0.0000	0.0000	0.0000	0.0000	0.0901	0.0000	0.0000
	avg	**0.0000**	0.0000	0.0000	0.0000	0.0000	0.0000	0.1056	0.0000	0.0000
**Leukemia**	std	**0.0000**	0.0000	0.0000	0.0000	0.0000	0.0000	0.0707	0.0000	0.0000
	avg	**0.0000**	0.0000	0.0000	0.0000	0.0000	0.0000	0.0000	0.0000	0.0000
**Brain_Tumor1**	std	**0.0316**	0.0502	0.0560	0.0546	0.0564	0.0735	0.0881	0.0574	0.0351
	avg	**0.0000**	0.0000	0.0000	0.0000	0.0500	0.0000	0.1111	0.0000	0.0000
**Brain_Tumor2**	std	**0.0000**	0.0000	0.0777	0.0831	0.0866	0.1235	0.1454	0.1235	0.0000
	avg	**0.0000**	0.0000	0.0000	0.0000	0.0000	0.0000	0.2083	0.0000	0.0000
**CNS**	std	**0.0000**	0.0883	0.0703	0.1179	0.1194	0.0856	0.1315	0.1365	0.0000
	avg	**0.0000**	0.0000	0.0000	0.0000	0.0000	0.0714	0.3333	0.0714	0.0000
**DLBCL**	std	**0.0000**	0.0000	0.0000	0.0395	0.0395	0.0395	0.1111	0.0000	0.0000
	avg	**0.0000**	0.0000	0.0000	0.0000	0.0000	0.0000	0.0625	0.0000	0.0000
**Leukemia1**	std	**0.0000**	0.0000	0.0000	0.0000	0.0000	0.0000	0.0602	0.0000	0.0000
	avg	**0.0000**	0.0000	0.0000	0.0000	0.0000	0.0000	0.0000	0.0000	0.0000
**Leukemia2**	std	**0.0000**	0.0000	0.0000	0.0395	0.0395	0.0527	0.0979	0.0452	0.0000
	avg	**0.0000**	0.0000	0.0000	0.0000	0.0000	0.0000	0.0625	0.0000	0.0000
**Lung_Cancer**	std	0.0158	0.0206	0.0234	0.0341	0.0363	0.0248	0.0463	0.0359	**0.0151**
	avg	0.0000	0.0000	0.0000	0.0000	0.0000	0.0476	0.0732	0.0238	**0.0000**
**Prostate_Tumor**	std	**0.0000**	0.0483	0.0422	0.0701	0.0844	0.0699	0.1589	0.0787	0.0000
	avg	**0.0000**	0.0000	0.0000	0.0000	0.0000	0.0500	0.3000	0.0955	0.0000
**Tumors_9**	std	**0.0000**	0.0703	0.0904	0.0000	0.0703	0.0811	0.2532	0.1309	0.0000
	avg	**0.0000**	0.0000	0.0000	0.0000	0.0000	0.0000	0.3667	0.0000	0.0000
**Tumors_11**	std	**0.0223**	0.0614	0.0211	0.0570	0.0488	0.0508	0.0638	0.0586	0.0369
	avg	**0.0000**	0.0000	0.0000	0.0000	0.0263	0.0557	0.1144	0.0588	0.0263
**Tumors_14**	std	0.0599	0.0516	**0.0603**	0.0719	0.0368	0.0559	0.0818	0.1008	0.0682
	avg	0.2624	0.2808	**0.1759**	0.2028	0.2713	0.2379	0.3906	0.2583	0.2284
ARV		**4.0786**	4.625	4.35	4.8393	5.0964	5.1571	7.4357	5.2857	4.1321
Rank		**1**	4	3	5	6	7	9	8	2

**Table A20:** Comparison of BISMA with other gene selection optimizers in terms of average fitness.

Datasets	Metrics	BISMA	BSMA	bGWO	BGSA	BPSO	bALO	BBA	BSSA	bWOA
**Colon**	std	**1.2910 × 10^−5^**	5.0206 × 10^−2^	1.1035 × 10^−1^	1.8282 × 10^−1^	1.1702 × 10^−1^	2.1096 × 10^−1^	1.3282 × 10^−1^	1.4476 × 10^−1^	4.1248 × 10^−5^
	avg	**2.5000 × 10^−5^**	1.1500 × 10^−3^	4.3875 × 10^−3^	9.8642 × 10^−2^	1.8077 × 10^−1^	1.0080 × 10^−1^	1.7705 × 10^−1^	8.0020 × 10^−2^	5.0000 × 10^−5^
**SRBCT**	std	**2.7403 × 10^−5^**	4.4567 × 10^−4^	3.3052 × 10^−4^	6.0770 × 10^−4^	3.7331 × 10^−4^	4.7086 × 10^−4^	5.4394 × 10^−2^	5.0897 × 10^−3^	4.1104 × 10^−5^
	avg	**6.4991 × 10^−5^**	7.2574 × 10^−4^	4.1594 × 10^−3^	1.9465 × 10^−2^	2.2162 × 10^−2^	2.1577 × 10^−2^	1.9757 × 10^−2^	2.3256 × 10^−2^	8.6655 × 10^−5^
**Leukemia**	std	**2.9568 × 10^−6^**	1.5407 × 10^−4^	2.8804 × 10^−4^	1.5587 × 10^−4^	2.2337 × 10^−4^	1.9186 × 10^−4^	3.8230 × 10^−2^	8.8001 × 10^−3^	6.4442 × 10^−6^
	avg	**7.0126 × 10^−6^**	2.5245 × 10^−4^	5.5505 × 10^−3^	2.1781 × 10^−2^	2.3520 × 10^−2^	2.3058 × 10^−2^	1.6518 × 10^−2^	2.4032 × 10^−2^	1.4025 × 10^−5^
**Brain_Tumor1**	std	3.0044 × 10^−2^	4.7527 × 10^−2^	5.3069 × 10^−2^	5.1816 × 10^−2^	5.3452 × 10^−2^	6.9773 × 10^−2^	6.3670 × 10^−2^	4.9713 × 10^−2^	**3.3378 × 10^−2^**
	avg	2.9561 × 10^−5^	9.4172 × 10^−4^	5.6841 × 10^−3^	2.2204 × 10^−2^	7.1128 × 10^−2^	2.3408 × 10^−2^	1.2274 × 10^−1^	2.4928 × 10^−2^	**2.5338 × 10^−5^**
**Brain_Tumor2**	std	8.2133 × 10^−6^	1.1604 × 10^−3^	7.3988 × 10^−2^	7.9166 × 10^−2^	8.2304 × 10^−2^	1.1744 × 10^−1^	1.3429 × 10^−1^	1.2498 × 10^−1^	**5.6840 × 10^−6^**
	avg	1.2057 × 10^−5^	7.5239 × 10^−4^	5.5899 × 10^−3^	2.2574 × 10^−2^	2.3715 × 10^−2^	2.3488 × 10^−2^	2.3165 × 10^−2^	1.4332 × 10^−2^	**1.2057 × 10^−5^**
**CNS**	std	**2.2179 × 10^−6^**	8.3687 × 10^−2^	6.6773 × 10^−2^	1.1206 × 10^−1^	1.1335 × 10^−1^	8.1427 × 10^−2^	1.5272 × 10^−1^	1.3596 × 10^−1^	2.2679 × 10^−5^
	avg	**7.0136 × 10^−6^**	2.2373 × 10^−3^	6.0843 × 10^−3^	2.3320 × 10^−2^	2.4165 × 10^−2^	9.1574 × 10^−2^	1.8163 × 10^−1^	9.2401 × 10^−2^	1.4027 × 10^−5^
**DLBCL**	std	**3.8548 × 10^−6^**	3.0615 × 10^−4^	2.1755 × 10^−4^	3.7526 × 10^−2^	3.7505 × 10^−2^	3.7552 × 10^−2^	6.1711 × 10^−2^	7.6159 × 10^−3^	9.0915 × 10^−6^
	avg	**9.1424 × 10^−6^**	3.7027 × 10^−4^	5.2249 × 10^−3^	2.1348 × 10^−2^	2.3149 × 10^−2^	2.2820 × 10^−2^	1.9112 × 10^−2^	2.4003 × 10^−2^	1.8285 × 10^−5^
**Leukemia1**	std	**7.6638 × 10^−6^**	2.3802 × 10^−4^	3.1146 × 10^−4^	3.6730 × 10^−4^	1.9525 × 10^−4^	2.9723 × 10^−4^	3.6838 × 10^−3^	1.0554 × 10^−2^	1.1873 × 10^−5^
	avg	**1.8772 × 10^−5^**	3.7545 × 10^−4^	5.1671 × 10^−3^	2.1616 × 10^−2^	2.3217 × 10^−2^	2.2705 × 10^−2^	1.9378 × 10^−2^	2.3827 × 10^−2^	3.2852 × 10^−5^
**Leukemia2**	std	**5.6343 × 10^−6^**	9.9607 × 10^−5^	2.0673 × 10^−4^	3.7572 × 10^−2^	3.7745 × 10^−2^	4.9938 × 10^−2^	5.3561 × 10^−2^	4.6911 × 10^−2^	5.3328 × 10^−6^
	avg	**1.1136 × 10^−5^**	2.4499 × 10^−4^	5.5479 × 10^−3^	2.2367 × 10^−2^	2.3699 × 10^−2^	2.3510 × 10^−2^	1.9595 × 10^−2^	2.4109 × 10^−2^	1.3363 × 10^−5^
**Lung_Cancer**	std	1.5004 × 10^−2^	1.9354 × 10^−2^	2.2153 × 10^−2^	3.2336 × 10^−2^	3.4492 × 10^−2^	2.3622 × 10^−2^	3.1687 × 10^−2^	4.1718 × 10^−2^	**1.4294 × 10^−2^**
	avg	5.1587 × 10^−5^	1.1885 × 10^−3^	6.1905 × 10^−3^	2.3317 × 10^−2^	2.4093 × 10^−2^	6.8815 × 10^−2^	6.3121 × 10^−2^	4.6873 × 10^−2^	**2.5794 × 10^−5^**
**Prostate_Tumor**	std	**6.7472 × 10^−6^**	4.6054 × 10^−2^	4.0020 × 10^−2^	6.6461 × 10^−2^	8.0247 × 10^−2^	6.6461 × 10^−2^	1.1415 × 10^−1^	7.7648 × 10^−2^	8.5258 × 10^−6^
	avg	**9.5157 × 10^−6^**	2.0126 × 10^−3^	6.1828 × 10^−3^	2.3454 × 10^−2^	2.4241 × 10^−2^	7.1027 × 10^−2^	1.0987 × 10^−1^	9.3377 × 10^−2^	1.4273 × 10^−5^
**Tumors_9**	std	8.9737 × 10^−4^	6.7888 × 10^−2^	8.5899 × 10^−2^	6.2023 × 10^−4^	6.6747 × 10^−2^	7.7087 × 10^−2^	1.9970 × 10^−1^	1.2797 × 10^−1^	**2.6422 × 10^−5^**
	avg	6.9857 × 10^−5^	1.5194 × 10^−3^	5.8854 × 10^−3^	2.2083 × 10^−2^	2.4162 × 10^−2^	2.3411 × 10^−2^	2.3214 × 10^−2^	2.4703 × 10^−2^	**2.6196 × 10^−5^**
**Tumors_11**	std	**2.1319 × 10^−2^**	5.7197 × 10^−2^	1.9912 × 10^−2^	5.4119 × 10^−2^	4.6341 × 10^−2^	4.8083 × 10^−2^	5.9891 × 10^−2^	5.7273 × 10^−2^	3.4943 × 10^−2^
	avg	**1.3923 × 10^−3^**	6.6026 × 10^−3^	6.4171 × 10^−3^	2.3604 × 10^−2^	4.9392 × 10^−2^	7.6599 × 10^−2^	1.2055 × 10^−1^	6.7590 × 10^−2^	2.6123 × 10^−2^
**Tumors_14**	std	5.5130 × 10^−2^	5.3345 × 10^−2^	**5.7317 × 10^−2^**	6.8032 × 10^−2^	3.5029 × 10^−2^	5.2960 × 10^−2^	7.0141 × 10^−2^	9.5849 × 10^−2^	6.4709 × 10^−2^
	avg	2.5180 × 10^−1^	2.7576 × 10^−1^	**1.7527 × 10^−1^**	2.1745 × 10^−1^	2.8210 × 10^−1^	2.5053 × 10^−1^	3.1859 × 10^−1^	2.7012 × 10^−1^	2.2178 × 10^−1^
ARV		**1.6964**	**1.6964**	3.7571	4.2286	5.9	7.1286	6.8143	6.8071	6.6429
Rank		**1**	**1**	3	4	5	9	8	7	6

**Table A21:** Comparison of BISMA with other gene selection optimizers in terms of average computational time.

Datasets	Metrics	BISMA	BSMA	bGWO	BGSA	BPSO	bALO	BBA	BSSA	bWOA
**Colon**	std	0.93215	0.55407	0.098098	0.11018	0.076472	0.069336	**0.19183**	0.23189	0.4158
	avg	79.1933	35.9194	14.2079	7.2215	4.2471	4.1295	**13.9446**	23.2622	26.0384
**SRBCT**	std	2.1619	0.51149	0.15702	0.11402	0.13534	0.16851	**0.25667**	0.3163	0.37599
	avg	93.6061	41.244	16.2856	8.8596	5.4073	5.2877	**16.3119**	27.1393	29.9446
**Leukemia**	std	7.2303	1.8022	0.3074	0.42745	0.27052	0.35454	**0.51794**	0.93854	1.2515
	avg	256.4992	122.7257	44.8501	23.5815	12.5313	12.2151	**45.0914**	79.2949	89.7865
**Brain_Tumor1**	std	6.9684	1.0527	0.278	0.45035	0.47769	0.32493	**0.49618**	1.069	1.2276
	avg	220.5351	103.2569	38.7039	21.6636	13.2493	12.7106	**40.4449**	68.416	74.6861
**Brain_Tumor2**	std	4.4718	2.0085	0.40876	0.4924	0.4705	0.34669	**0.57963**	1.4683	1.9697
	avg	354.245	176.7797	63.4666	29.9924	13.3176	12.3337	**60.1049**	110.4912	131.5993
**CNS**	std	4.7455	1.4788	0.51911	0.26787	0.19856	0.2563	**0.6056**	0.94437	1.1606
	avg	248.504	122.8625	44.5969	22.0423	10.8899	10.3101	**43.5037**	77.9093	89.9202
**DLBCL**	std	5.3919	0.93684	0.23064	0.16063	0.29357	0.16353	**0.47267**	0.61759	1.1042
	avg	200.6234	94.9785	35.3048	18.7326	10.6001	10.3269	**35.7383**	61.9793	69.2286
**Leukemia1**	std	4.3623	1.1156	0.31638	0.35829	0.27141	0.19161	**0.52207**	0.86822	0.85614
	avg	194.386	92.0658	34.0793	17.7135	9.9582	9.5621	**34.4391**	60.048	66.7794
**Leukemia2**	std	7.374	3.0726	0.49382	0.61819	0.5241	0.50114	**0.61738**	1.9491	2.7261
	avg	399.2129	192.557	69.7935	36.9327	19.1668	18.0316	**69.7835**	123.9545	144.0557
**Lung_Cancer**	std	41.6799	4.5389	1.0736	3.7226	4.3019	3.6112	**4.3848**	2.089	2.7099
	avg	515.1456	233.6435	99.2504	93.316	77.8452	75.9999	**127.7409**	190.8128	167.6969
**Prostate_Tumor**	std	17.4231	2.7956	0.56853	0.55518	0.82953	0.5596	**1.1379**	1.3348	1.8037
	avg	383.2946	183.4421	68.6138	42.0024	25.8001	25.2016	**72.0151**	122.9783	133.436
**Tumors_9**	std	2.9367	1.1039	0.25397	0.356	0.42194	0.23273	**0.39294**	0.77615	1.0106
	avg	203.6074	98.814	36.386	18.1879	9.237	8.8404	**35.6073**	62.8321	71.9579
**Tumors_11**	std	11.6164	3.5569	1.045	2.7062	2.8401	3.2718	**3.7758**	1.9198	1.6793
	avg	465.5284	226.3375	93.2904	78.8383	61.7486	60.114	**113.7264**	175.511	163.5641
**Tumors_14**	std	78.3081	9.9354	**2.1979**	13.6846	7.0692	10.4616	8.8472	4.4011	5.6434
	avg	664.032	309.3361	**159.1758**	202.5748	176.6571	176.1249	235.2403	308.2242	212.441
ARV		9	7.95	4.1857	3.1	1.8929	**1.2571**	4.6643	6.2643	6.6857
Rank		9	8	4	3	2	**1**	5	6	7
